# A novel A2a adenosine receptor inhibitor effectively mitigates hepatic fibrosis in a metabolic dysfunction-associated steatohepatitis mouse model

**DOI:** 10.7150/ijbs.92371

**Published:** 2024-03-03

**Authors:** Seojeong Park, Seohui Hwang, Jingyang Sun, Kyung-Hwa Jeon, Naeun Sheen, Sumin Shin, Tae Hyun Kim, Young-Sun Lee, Wonhyo Seo, Jae-Sang Ryu, Youngjoo Kwon

**Affiliations:** 1 College of Pharmacy, Ewha Womans University, 52 Ewhayeodae-gil, Seodaemun-gu, Seoul, 03760, Republic of Korea.; 2 Muscle Physiome Research Center and Research Institute of Pharmaceutical Sciences, College of Pharmacy, Sookmyung Women's University, Seoul, 04310, Republic of Korea.; 3 Department of Internal Medicine, Korea University College of Medicine, Seoul, 08308, Republic of Korea.

**Keywords:** A2aAR, hepatic fibrosis, MASH, ZM241385, novel A2aAR antagonist RAD11 [4-(6-(methylamino)-2-(phenylethynyl)-9H-purin-9-yl)phenol]

## Abstract

Hepatic fibrosis exacerbates mortality and complications in progressive metabolic dysfunction-associated steatohepatitis (MASH). The role of the adenosine 2A receptor (A2aAR) in hepatic fibrosis within the context of MASH remains uncertain. This study aims to elucidate the involvement of the A2aAR signaling pathway and the efficacy of a novel potent A2aAR antagonist in treating hepatic fibrosis in MASH-induced mice fed a chlorine-deficient, L-amino acid-defined, high fat diet (CDAHFD). A2aAR overexpression in LX-2 cells increased fibrosis markers, whereas the known A2aAR antagonist, ZM241385, decreased these markers. A novel A2aAR antagonist, RAD11, not only attenuated fibrosis progression but also exhibited greater inhibition of the A2aAR signaling pathway compared to ZM241385 in mice with MASH, activated primary hepatocytes, and LX-2 cells. RAD11 exhibited a dual antifibrotic mechanism by targeting both activated HSCs and hepatocytes. Its superior antifibrotic efficacy over ZM241385 in the MASH condition stems from its ability to suppress A2aAR-mediated signaling, inhibit HSC activation, reduce hepatic lipogenesis in hepatocytes, and mitigate lipid accumulation-induced oxidative stress-mediated liver damage. This study has shed light on the relationship between A2aAR signaling and hepatic fibrosis, presenting RAD11 as a potent therapeutic agent for managing MASH and hepatic fibrosis.

## Introduction

Metabolic dysfunction-associated fatty liver disease (MASLD, also kwon as an non-alcoholic fatty liver disease, NAFLD) is currently the most prevalent liver disease in Western countries, and its incidence is on the rise globally 1. Histological changes in MASLD are similar to alcoholic fatty liver disease, but they occur in individuals without a history of alcohol abuse 2. Instead, the main risk factors for MASLD are related to lifestyle, such as high-carbohydrate and high-fat diets and sedentary behavior 3. MASLD is a spectrum of liver diseases that can range from simple steatosis to more severe forms of the disease, such as metabolic dysfunction-associated steatohepatitis (MASH), fibrosis, cirrhosis, and even hepatocellular carcinoma. Unlike simple steatosis, where fat accumulates in the liver without inflammation or liver cell injury, MASH is a more severe form of the disease characterized by hepatic steatosis, hepatocellular ballooning, and lobular inflammation, in which fat accumulation in the liver is accompanied by inflammation and liver cell injury 4-6. These features are significant enough to be used as histological parameters for the NAFLD activity score (NAS), which assesses the severity of MASLD in patients 7.

As MASH progresses, it is often accompanied by hepatic fibrosis, which is a major cause of increased liver-related mortality and complications 8-10. In addition, the severity of hepatic fibrosis is a crucial determinant of the long-term prognosis of MASLD patients, regardless of other histological features and NAS 10. Therefore, the development of an effective MASH treatment that can inhibit fibrosis, which would decrease the mortality rate of MASLD, is of utmost importance 11. Currently, there are no U.S. Food and Drug Administration (FDA)-approved drugs for treatment of MASH. Accordingly, MASH-related research is being actively conducted worldwide to develop therapies for MASLD. MASH is considered a progressive form of MASLD, in which fat accumulates in the liver and causes inflammation and damage to liver cells. As the disease progresses, hepatic stellate cells (HSCs) are activated and begin to produce excess collagen, leading to hepatic fibrosis. Proinflammatory and fibrogenic cytokines or mediators released from various hepatic cells, including Kupffer cells, contribute to the activation of HSCs 12-14. In MASH, Kupffer cells, specialized macrophage in the liver, become activated and produce proinflammatory cytokines, such as tumor necrosis factor-alpha (TNF-α) and interleukin-6 (IL-6), leading to promotion of liver inflammation and fibrosis. In addition to Kupffer cells, other cells in the liver, such as hepatocytes, endothelial cells, and immune cells, also release proinflammatory cytokines and chemokines that contribute to the development and progression of MASH. The lipid accumulation in hepatocytes, oxidative stress, and mitochondrial dysfunction are among the factors that trigger the release of these inflammatory mediators 15-17.

Circulating adenosine and adenosinergic signaling have been implicated in the development and progress of liver injury and hepatic fibrosis 18. Adenosine is a signaling molecule generated from the breakdown of ATP, which is released by various cell types in response to stimuli such as injury or inflammation. In the liver, parenchymal hepatocytes and endothelial cells are the primary sources of ATP release in response to injury or stress. The extracellular conversion of ATP to ADP, AMP, and adenosine is mediated by enzymes such as CD39 and CD73, which are expressed on the surface of various cell types in the liver 19. Extracellular adenosine exerts its effects by binding to specific receptors, including adenosine 1 receptor (A1AR), adenosine 2A receptor (A2aAR), adenosine 2B receptor (A2bAR), and adenosine 3 receptor (A3AR). Chan et al. have shown that, of the four adenosine receptors, increased adenosine caused by liver injury primarily acts on A2aAR, especially on the transmembrane of HSCs, leading to an increase in the expression of collagen Ⅰ and Ⅲ, which are critical for inducing hepatic fibrosis 20.

The role of A2aAR in triggering hepatic fibrosis is a subject of controversy. Several liver injury models have shown antifibrotic effects in the liver when A2aAR is deficient 20. Additionally, the A2aAR selective antagonist ZM241385 has been found to attenuate hepatic fibrosis in mice exposed to tetrachloromethane (CCl_4_) or thioacetamide (TAA). *In vivo* studies have also demonstrated that caffeine, a non-selective antagonist of A2aAR, has antifibrotic effects in liver injury models 20, 21. On the other hand, the A2aAR signaling pathway can inhibit the release of proinflammatory cytokines by acting on macrophages as well 19. Likewise, A2aAR-deficient mice maintained the release of proinflammatory cytokines when lipopolysaccharide (LPS) was administered 22. Jing et al. also showed that MASH was worse in A2aAR-disrupted mice compared to control mice 23. These conflicting results suggest that the role of A2aAR in hepatic fibrosis is complex and context-dependent. Although A2aAR activation may promote fibrosis by stimulating collagen production in HSCs, it may also have anti-inflammatory effects that could limit fibrosis. Further research is needed to fully elucidate the mechanisms underlying the effects of adenosinergic signaling on hepatic fibrosis and to determine the therapeutic potential of targeting this pathway.

Therefore, this study aimed to confirm the role of A2aAR in the process of hepatic fibrogenesis and to evaluate whether A2aAR could be a meaningful therapeutic target for hepatic fibrosis and MASH. Herein, we report the discovery and characterization of RAD11, a novel potent A2aAR antagonist, which attenuated hepatic fibrosis by blocking the A2aAR downstream signaling of the cAMP/PKA/CRE pathway.

## Materials and methods

### RAD11 synthesis

RAD11 was synthesized and found to be 97% pure by high-performance liquid chromatography. Additional information on the synthesis, the ^1^H and ^13^C nuclear magnetic resonance spectral data and liquid chromatography-high resolution mass spectroscopic data for RAD11, can be found in the Supplementary Information (**Scheme S1, Figs. S1 and S2**).

### CDAHFD-induced murine model of MASH

Six-week-old male C57BL/6J mice obtained from Raon-bio (Yongin-si, South Korea) were housed in a temperature-controlled room with 12 h light/dark cycle and randomly assigned two groups. To investigate CDAHFD-induced MASH and hepatic fibrosis, mice were fed either a normal chow diet or a CDAHFD (#A06071302, Research Diets, NJ, USA) for 6 weeks. To evaluate the efficacy of A2aAR agonists, 2 weeks after initiating CDAHFD, the mice in the CDAHFD group were intraperitoneally injected with vehicle, ZM241385, or RAD11 at a dose of 20 mg/kg every other day for 5 weeks. After sacrifice, liver tissue samples were fixed in a 4% paraformaldehyde solution and embedded in paraffin for staining or frozen at -70 ℃. Serum samples were obtained by centrifuging blood collected from the caudal vena cava (CVC). All animal experiments were approved by the Institutional Animal Care and Use Committee at Ewha Womans University (Seoul, South Korea) (approval No. 19-042) in accordance with guidelines for animal welfare and ethical conduct.

### Liver function tests

To measure the total concentration of serum aspartate aminotransferase (AST) and alanine aminotransferase (ALT), serum samples were analyzed using a chemistry analyzer (Hitachi 7020, Tokyo, Japan) following the manufacturer's instructions.

### LX-2 cell culture and plasmid transfection

LX-2 cells were cultured in Dulbecco's modified Eagle's medium supplemented with 10% fetal bovine serum and 1% penicillin-streptomycin. For transfection, 5×10^5^ LX-2 cells were seeded and, after 24 h, were transfected with the CRE-pGL3 vector alone or the pcDNA4-myc-His A vector fused with h*ADORA2A* using Lipofectamine 2000 reagent (Invitrogen, CA, USA) in serum-free medium for 6 h. For knockdown of the h*ADORA2A* or h*ADORA2B*, all siRNAs were synthesized by Bioneer (Bioneer Corp., Korea) and transfected into LX-2 cells for 24 h. The medium was then replaced with serum-free medium containing 10 μM of each compound and incubated for 18 h.

### Primary hepatocyte culture and PA:OA treatment

Primary hepatocytes were isolated from C57BL/6J mice and immediately seeded in 6-well plates. When approximately 80% confluency was reached, a mixture of palmitic acid and oleic acid (PA:OA) was treated at final concentrations of 0.5 mM (#P0500, Sigma-Aldrich, US) and 1 mM (#O1008, Sigma-Aldrich, US), respectively. ZM241385 and RAD11 (10 μM of each) were added individually with PA:OA, and cells were harvested after 12 h. For this experiment, 5 mM stocks of fatty acid solutions were prepared as described previously 24.

### Immunofluorescence of primary HSCs

Mouse liver HSCs were isolated by in situ collagenase perfusion and differential centrifugation on OptiPrep (Sigma-Aldrich, US) density gradients. HSCs were treated with transforming growth factor beta 1 (TGF-β1, R&D Systems, Inc., US) and RAD11 with medium replacement. Activated HSCs (on day 5 cultured cells) were fixed, cells were fixed with 4% paraformaldehyde and permeabilized with 0.1% Triton X-100 in PBS. HSCs were then incubated overnight with the α-smooth muscle actin (α-SMA, GeneTex, US) antibody. After washing with PBS, HSCs were incubated with Alexa Fluor 488 (Abcam, Cambridge, UK) for 1 h and counterstained with 1 μg/mL of DAPI. The slides were evaluated at 200× magnification using Nikon A1R confocal microscope (Nikon, Tokyo, Japan).

### Quantitative real-time polymerase chain reaction (qRT-PCR)

A qRT-PCR assay was performed as described previously 25. Briefly, to extract RNA from liver tissues, 1 mg of liver tissue was homogenized using TissueLyser II (QIAGEN, Hilden, Germany) in Tri-RNA reagent (FAVORGEN Biotech Crop., Kaohsiung, Taiwan). The extracted RNA was reverse-transcribed to complementary DNA (cDNA), and quantitative gene expression analysis was performed using a SensiFAST SYBR No-ROX kit (Bioline, London, UK). The qRT-PCR was conducted using a CFX96^TM^ real-time PCR system (Bio-Rad, CA, USA). The qPCR amplification was performed as follows: polymerase activation for 2 min at 95 °C, followed by 30 cycles of 10 sec at 95 °C, 30 sec at the median *T_m_* of each primer, and 20 sec at 72 °C. The relative mRNA quantity was determined using the *ΔΔ*Ct method and was normalized by *GAPDH* expression. All reactions were performed in triplicate.

### Luciferase reporter gene assay

The activity of the CRE promoter was evaluated according to a method reported previously 26. The predicted CRE promoter region was cloned into the pGL3-basic vector (Promega, WI, USA) along with firefly and Renilla luciferase reporters. HEK293 cells (5×10^5^) were seeded in 60-mm culture dishes and transfected with 0.5 μg of pGL3-CRE vector alone or in combination with pcDNA4-myc-His A vector fused with *ADORA2A*. After 6 h, the culture medium was replaced with serum-free medium containing 10 μM of ZM241385, CGS21680, or each compound, followed by incubation for 18 h. The cells were then lysed with reporter lysis buffer, and the relative luciferase activity was determined using a Luciferase Assay system (#E2940, Promega, WI, USA) according to the manufacturer's protocol with an Infinite M200 PRO Microplate Reader (Tecan Group Ltd., Männedorf, Switzerland), equipped at Ewha Drug Development Research Core Center. The luciferase activity was normalized using Renilla luciferase as an internal control to account for the process of transfection itself.

### Real-time monitoring of primary HSC morphology changes

After primary HSCs were isolated from C57BL/6J mice, they were seeded in 12-well plates. After 12 h, the HSCs were treated with either TGF-β1, ZM241385, or RAD11. From day 2 to day 5, an xCELLigence RTCA HT system (Agilent, CA, USA) was used to perform real-time cell morphology analysis. The system continuously monitored HSCs and recorded changes in cellular impedance, which correlates with alterations in cell morphology. Measurements were performed every 30 min, providing dynamic insights into the response of HSCs to the respective treatments. The collected real-time data were arranged in chronological order, and a video representation of the changes in HSC morphology was generated.

### Statistical analysis

Statistical analyses were conducted using GraphPad Prism statistical software (Version 6.01, GraphPad Software Inc, CA, USA). Data are presented as mean ± standard deviation of at least triplicate experiments. To compare two data sets, an unpaired Student's t-test was used. Multiple comparisons were analyzed by one-way analysis of variance (ANOVA), followed by Tukey's post hoc test. The homogeneity of variances was tested before ANOVA. Post hoc tests were performed only if F achieved *p*<0.05 and there was no significant variance inhomogeneity. Data were normalized to each matched control value and were analyzed with a non-parametric statistical test, the Kruskal-Wallis test, followed by Dunn's multiple comparison test. All calculated *p* values were two-sided, and a *p*-value less than 0.05 was considered statistically significant for all analyses.

For further details regarding *in vivo* immunohistochemistry assay, Western blot analysis, cAMP assay, and gene set enrichment analysis, please refer to the Supplementary information. The antibodies and the primary and siRNA sequences used for this study were listed in Table S1 and S2, respectively.

## Results

### A2aAR signaling pathway correlates with hepatic fibrosis in a murine model of MASH

**Figure 1:**
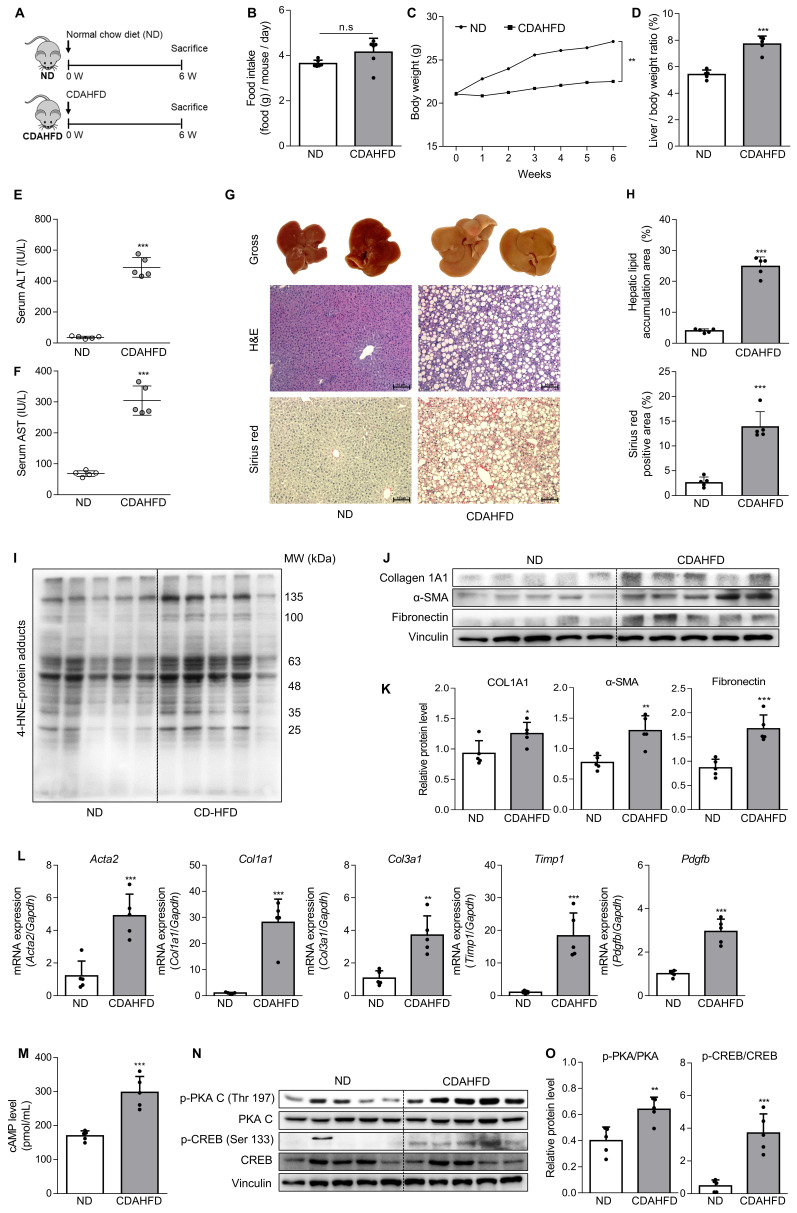
** The A2aAR signaling pathway correlates with hepatic fibrosis in a murine MASH model.** (A) Schematic overview of the animal experiment. Male C57BL/6J mice were fed a normal chow diet (ND) (n = 5) or a chlorine-deficient, L-amino acid-defined, high-fat diet (CDAHFD) (n = 5) for 6 weeks. (B, C) Food intake and body weight changes in mice of ND- and CDAHFD groups. (D, E) Liver-to-body weight ratio (%) and (E, F) serum ALT and AST levels in mice of ND- and CDAHFD groups. (G, H) Representative images of gross liver and H&E and Sirius red staining of liver sections. Stained histological sections were quantified (100× magnification. Scale bars, 10 μm). (I-L) Liver tissue lysates were subjected to western blotting and RT-qPCR analyses. (M) The total concentration of cAMP in the serum of mice was evaluated. (N, O) Liver tissue samples were subjected to western blotting and quantification. Statistical analysis was performed using an unpaired Student's t-test. *** *p* < 0.001, ** *p* < 0.01, * *p* < 0.05, versus ND group. n.s: nonsignificant.

To confirm the association between the A2aAR signaling pathway and hepatic fibrosis that occurs with the progression of MASH, a murine model of MASH was developed in CDAHFD-fed mice for 6 weeks **(Fig. 1A)**. A control group was fed a normal chow diet (ND) for the same period. Although there was no significant difference in food intake between the two groups (**Fig. 1B**), the body weights of the CDAHFD group were slightly lower than those of the ND-fed mice (**Fig. 1C**). The CDAHFD group exhibited features of hepatic fibrosis induced by MASH, including increased liver-to-body weight ratio (**Fig. 1D**), elevated serum ALT and AST levels (**Fig. 1E and F**), and histological evidence of increased lipid accumulation and collagen deposition (**Fig. 1G and H**). The expression levels of protein adducts formed by 4-hydroxy-2-nonenal (4-HNE), a well-known marker of oxidative stress and a trigger of liver diseases such as MASH 27, 28 were also higher in the CDAHFD-fed group (**Fig. 1I**). Moreover, the protein expressions of α-SMA, a marker of HSC activation, and collagen and fibronectin, profibrotic markers, were significantly increased in the CDAHFD group (**Fig. 1J and K**). The mRNA levels of profibrotic genes were also higher in the MASH-induced group (**Fig. 1L**). These results indicate that CDAHFD-fed mice were a well-established mouse model of MASH-induced hepatic fibrosis. Considering that the binding of adenosine to A2aAR or A2bAR increases intracellular cAMP levels through the activation of adenylyl cyclase, and that cAMP can be transported out and extracellular cAMP serves as a significant source of extracellular adenosine, we measured serum cAMP concentration 21, 29. Interestingly, serum cAMP concentration (**Fig. 1M**) and the protein levels of the activated forms of protein kinase A (PKA) and cAMP response element-binding (CREB), which are downstream factors of A2aAR (**Fig. 1N and O**), were remarkably upregulated in this MASH model. The MASH patient liver showed higher expression of p-PKA and p-CREB compared to healthy individuals (**Fig. S3A**). Gene Set Enrichment Analysis (GSEA) using GEO dataset (GSE63067) also indicated a higher enrichment score in cAMP/PKA/CREB signaling pathway-related gene set in the MASH group compared to the healthy group (**Fig. S3B**). Taken together, these findings suggest that A2aAR downstream factors are upregulated when MASH is induced *in vivo*, indicating that the A2aAR signaling pathway is correlated with MASH-induced hepatic fibrosis.

### Activation of HSCs and the A2aAR signaling pathway is most likely responsible for MASH-induced hepatic fibrosis

**Figure 2:**
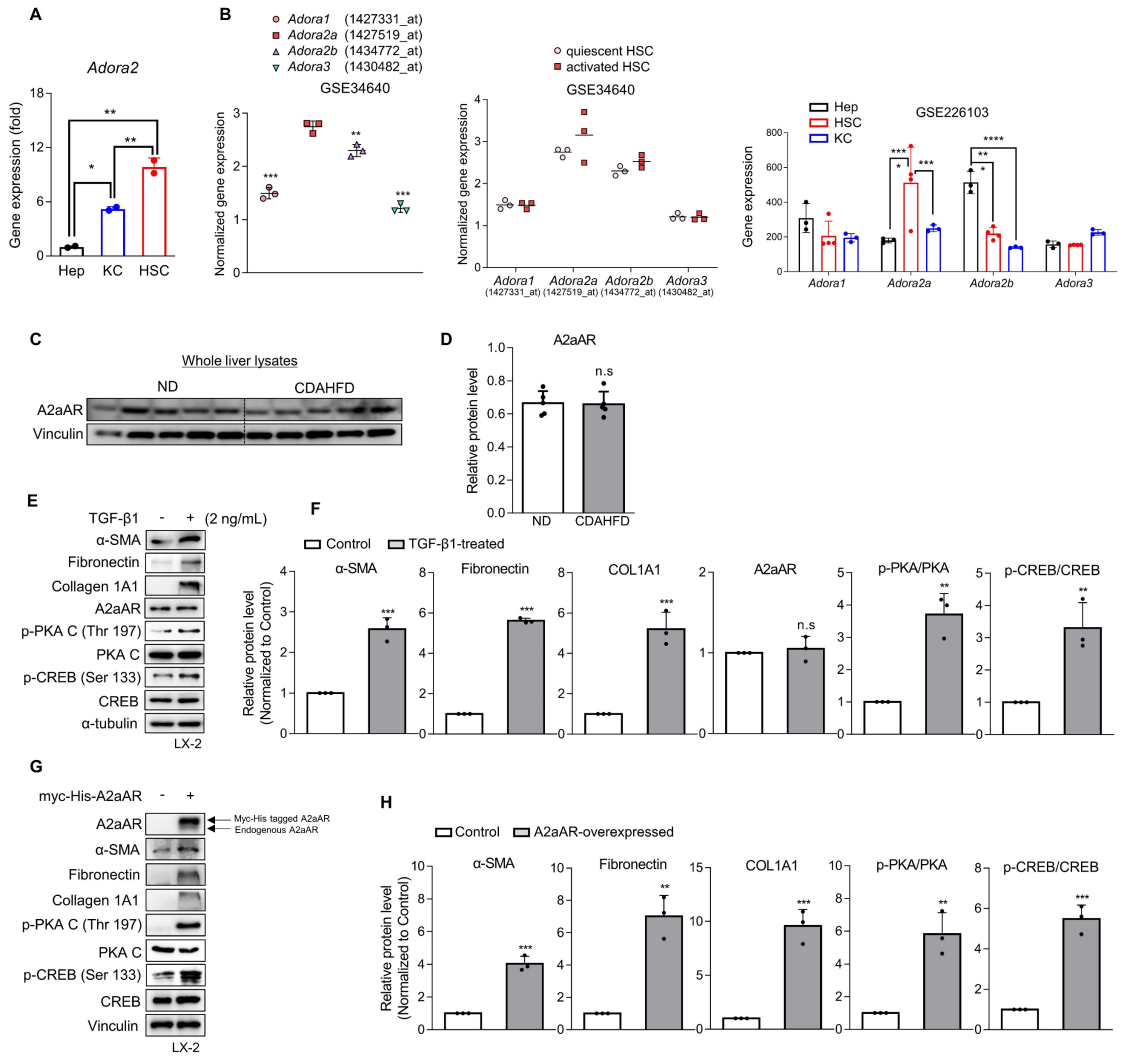
** Activation of HSCs and the A2aAR signaling pathway is most likely responsible for MASH-induced hepatic fibrosis.** (A) Freshly isolated primary hepatocytes, Kupffer cells (KC) and HSCs were subjected to RT-qPCR analyses. (B) GSE34640 for mouse primary HSCs and GSE226103 for mouse hepatocytes (Hep), HSCs, and KC were used for evaluating the gene expression levels of four adenosine receptors (n=3 per group). (C, D) Protein expression level of A2aAR in liver tissues was evaluated using western blotting and quantified. (E, F) LX-2 cells were activated by treatment with 2 ng/mL TGF-β1 for 24 h and evaluated using western blotting. (G, H) LX-2 cells were transfected with pcDNA4-myc-His-h*ADORA2A* for 6 h, followed by incubation for 18 h to overexpress A2aAR. Transfected cells were assessed using western blotting analysis. *** *p* < 0.001, ** *p* < 0.01, * *p* < 0.05. n.s: nonsignificant.

Various types of hepatic cells are involved in the process of MASH-induced hepatic fibrosis 12-14. To investigate which hepatic cell type is most likely responsible for MASH-induced hepatic fibrosis through A2aAR signal activation, we analyzed the gene expression of A2aAR in primary Kupffer cells, hepatocytes, and HSCs isolated from C57BL/6J mice (**Fig. 2A**). HSCs were found to be the most influential hepatic cell type, which is consistent with the knowledge that HSCs significantly contribute to the fibrogenic response in the liver by transdifferentiating into myofibroblasts 30. Based on this finding, we compared the gene expressions of the four adenosine receptors in quiescent and activated mouse primary HSCs from the GEO datasets (GSE34640 and GSE226103) 31, showing that A2aAR was the most highly upregulated in activated HSCs (**Fig. 2B**). However, there was no significant difference in A2aAR expression between the ND and CDAHFD groups in whole liver lysate (**Fig. 2C and D**). Therefore, to conduct further HSC-specific studies by mimicking the situation of hepatic fibrogenesis *in vitro*, LX-2 cells, a human hepatic stellate cell line, were treated with TGF-β1. TGF-β1 is regarded as a major cytokine that contributes to the development of fibrosis in various tissues 32. The results showed that addition of TGF-β1 upregulated α-SMA, fibronectin, and collagen. The protein expression of A2aAR was constant regardless of whether LX-2 cells were activated, but the activated forms of A2aAR downstream factors were significantly increased (**Fig. 2E and F**). To verify whether the increase in p-PKA and p-CREB levels was due to A2aAR itself, we overexpressed A2aAR in LX-2 cells using the pcDNA4-myc-His A vector plasmid fused with h*ADORA2A* (**Fig. 2G**). The trends in expression of A2aAR downstream factors and fibrogenesis markers in A2aAR-overexpressing LX-2 cells were similar to those in LX-2 cells activated by TGF-β1 to mimic hepatic fibrogenesis (**Fig. 2H**). These results suggest that activated HSCs and A2aAR-overexpressing HSCs induce hepatic fibrosis, and PKA and CREB are activated under these conditions. These findings indicate that hepatic fibrogenesis can occur through an activated A2aAR signaling pathway.

### A2aAR antagonist ZM241385 exerts an antifibrotic effect on activated HSCs

**Figure 3:**
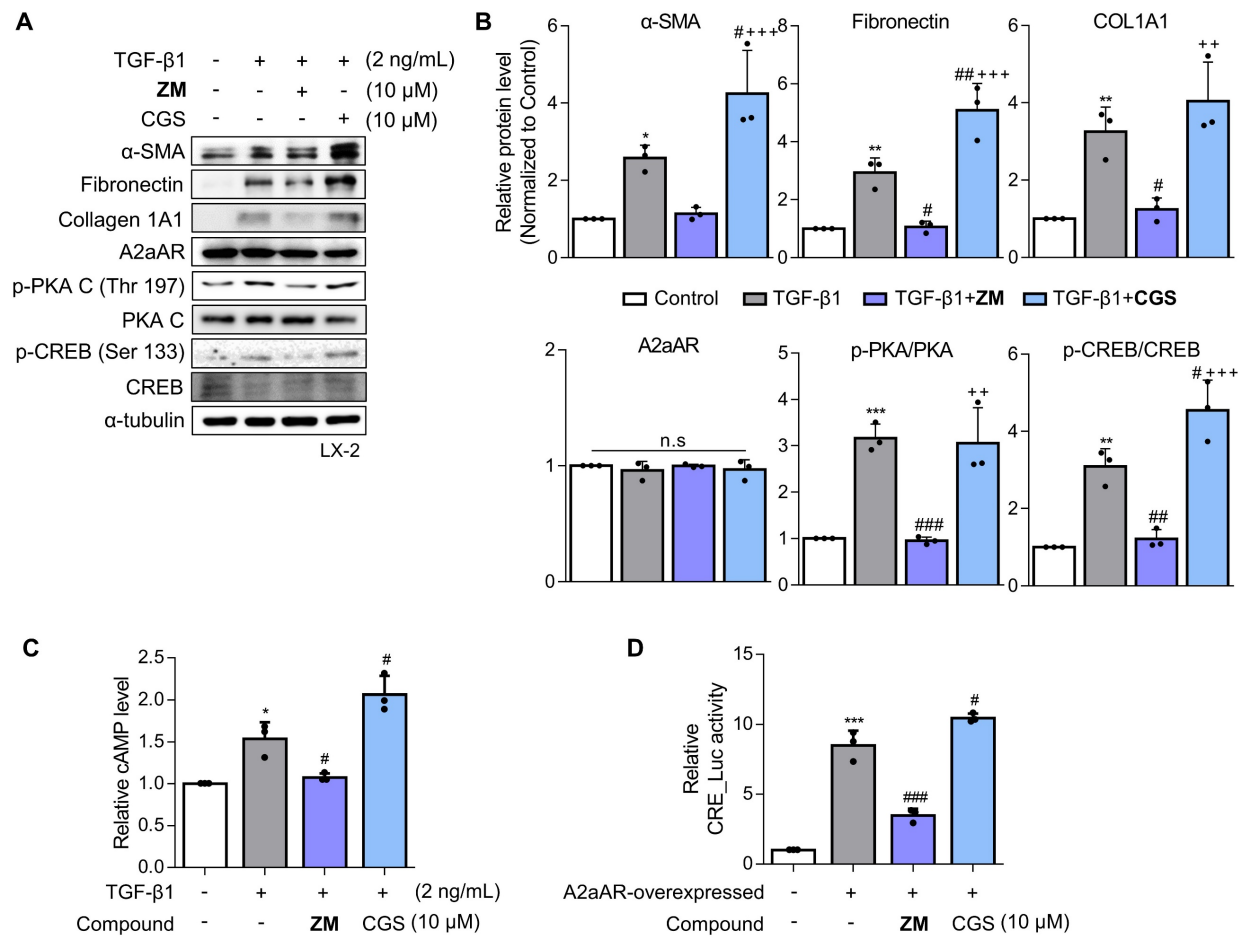
** ZM241385, an A2aAR-specific antagonist, but not CGS21680 exerts an antifibrotic effect on activated HSCs.** (A, B) LX-2 cells, activated with 2 ng/mL TGF-β1 for 6 h, were treated with 10 μM of either ZM241385 or CGS21680 for 18 h. Cultured LX-2 cells were collected and subjected to western blotting analysis, and protein expression was quantified. (C) The total concentration of cAMP was measured in LX-2 cells incubated with 2 ng/mL TGF-β1 for 6 h, followed by incubation with 10 μM of either ZM241385 or CGS21680 for 18 h. (D) The activity of the CRE promoter was analyzed in HEK293 cells transfected with 0.5 μg of the pGL3-CRE alone or in combination with pcDNA4-myc-His A vector fused with h*ADORA2A* vector, followed by incubation with 10 μM of ZM241385 or CGS21680 for 18 h. Statistical analyses were performed using one-way ANOVA. *** *p* < 0.001, ** *p* < 0.01, * *p* < 0.05 versus control group; ### *p* < 0.001, ## *p* < 0.01, # *p* < 0.05 versus only TGF-β1-treated group; ⁺⁺⁺ *p* < 0.001, ⁺⁺ *p* < 0.01 versus ZM241385-treated group.

Since a positive correlation among A2aAR signaling, HSC activation, and hepatic fibrosis was demonstrated, we hypothesized that A2aAR selective antagonists might reduce hepatic fibrosis. We treated LX-2 cells with the well-known selective A2aAR antagonist ZM241385 as a positive control and with the specific A2aAR agonist CGS21680 33. Under the same HSC activation conditions as shown in Figure 2, ZM241385 resulted in lower protein expressions of α-SMA, fibronectin, and collagen compared to LX-2 cells treated only with TGF-β1. In contrast, CGS21680 increased the expression of these proteins (**Fig. 3A and B**). Additionally, ZM241385 decreased the expression of the downstream factors of A2aAR, such as p-PKA and p-CREB. Even though ZM241385 and CGS21680 did not affect the expression of the A2aAR protein, changes in cAMP level confirmed that changes in the protein levels of A2aAR downstream factors were due to altered A2aAR activity rather than its expression (**Fig. 3C**). Moreover, ZM241385 impeded the A2aAR signaling pathway and, therefore, reduced the activity of the CRE promoter (**Fig. 3D**). Overall, our findings suggest that ZM241385 has an antifibrotic effect on the intracellular milieu by inhibiting the A2aAR signaling pathway.

### RAD11 mitigates the severity of hepatic damage and fibrosis in a mouse model of MASH

**Figure 4:**
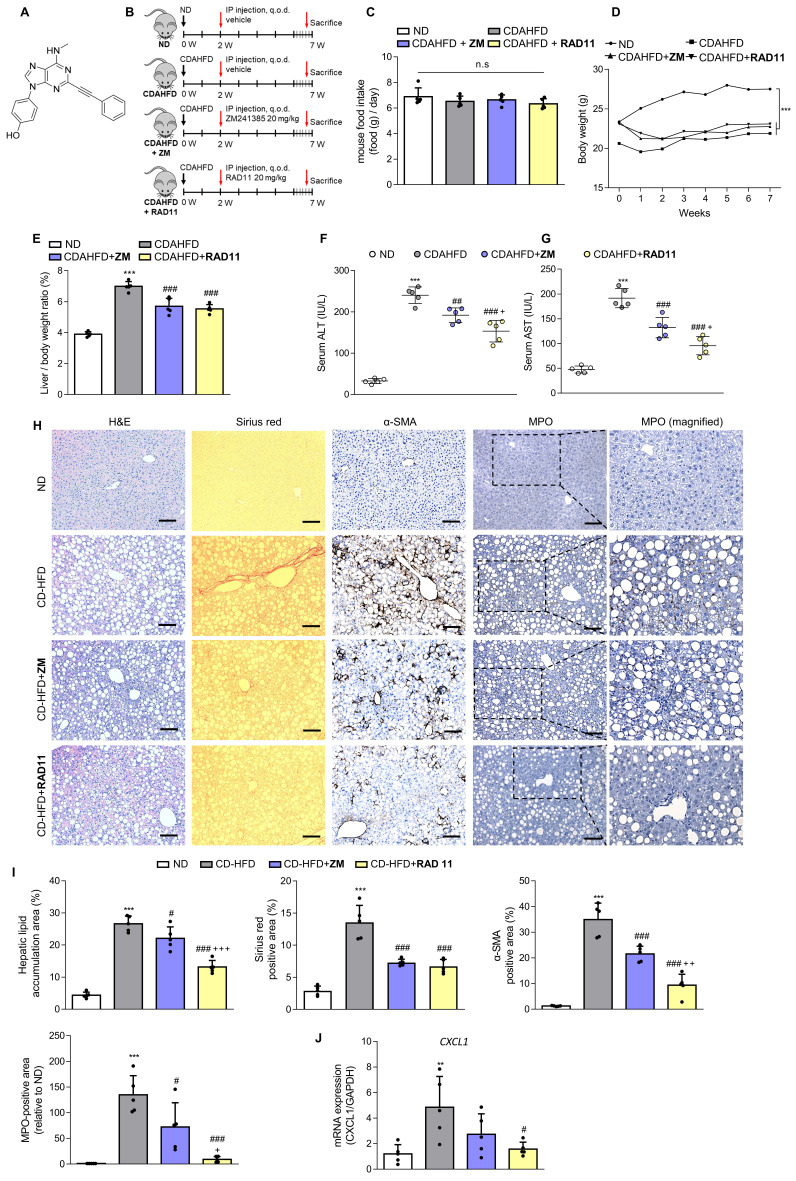

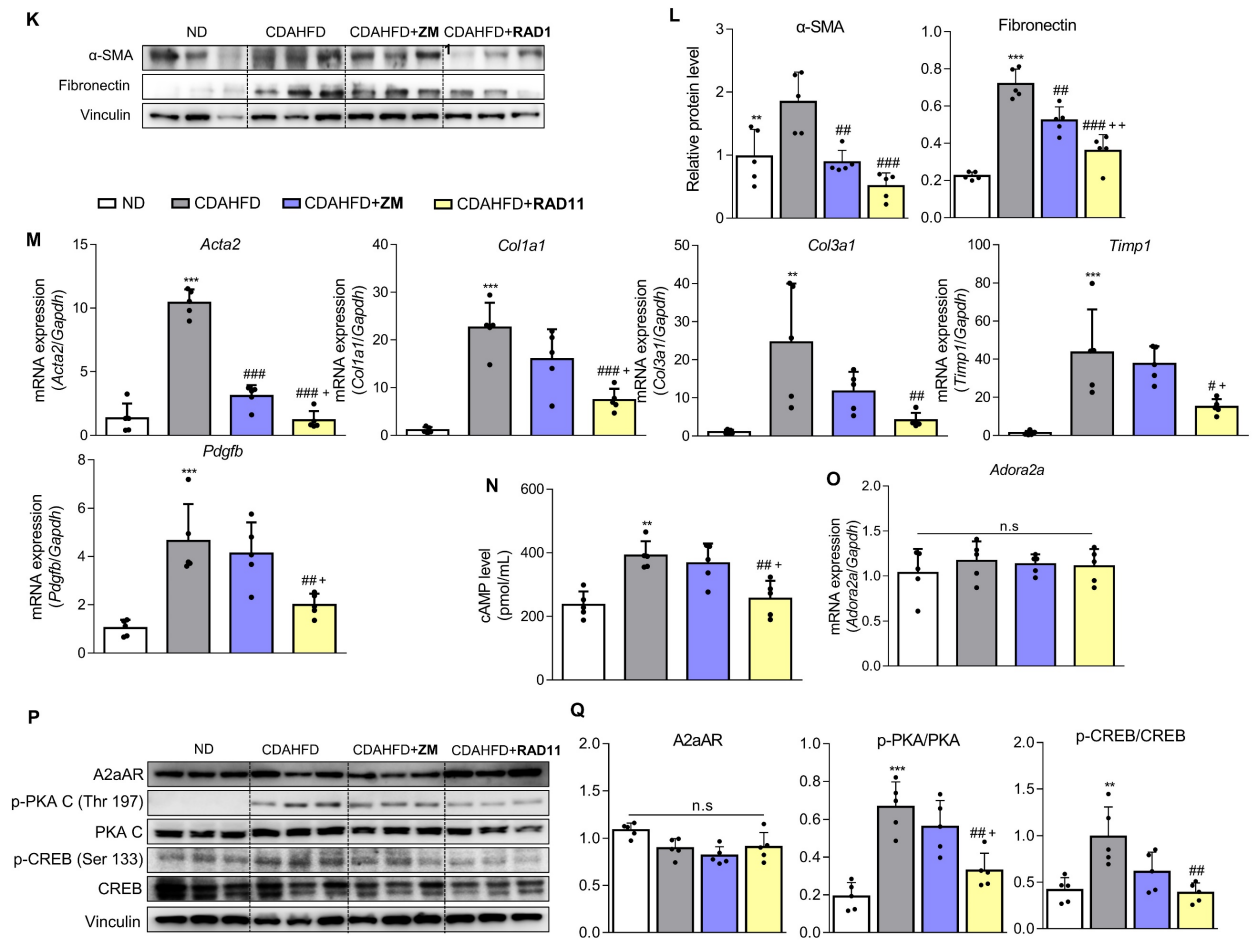
** RAD11 mitigates the severity of hepatic damage and fibrosis in a MASH mouse model.** (A) Chemical structure of adenine derivative RAD11. (B) Experimental scheme of animal experiments. Intraperitoneal injections were performed every other day for 5 weeks. (C, D) Food intake and body weight of each group. (E) Liver-to-body weight ratio (%). (F, G) Serum ALT and AST levels. (H, I) Liver tissue sections were subjected to IHC staining (100× magnification. Scale bars, 10 μm) and quantified. Representative images of H&E, Sirius red, α-SMA, and MPO staining of liver sections from each group. (J, M, and O) mRNA expressions in liver tissues extracted from mice in each group were assessed by RT-qPCR analysis (K, L and P, Q). Protein expressions in liver tissue lysates were evaluated by western blotting and quantified. (N) The total concentration of cAMP in liver sections from each group. Statistical analyses were performed using ordinary one-way ANOVA. *** *p* < 0.001, ** *p* < 0.01 versus ND group; ### *p* < 0.001, ## *p* <0.01, # *p* < 0.05 versus CDAHFD group; ⁺⁺⁺ *p* < 0.001, ⁺⁺ *p* < 0.01, ⁺ *p* < 0.05 versus ZM241385-treated group. n.s: nonsignificant.

After confirming the potential of ZM241385 in the treatment of hepatic fibrosis in MASH-induced mice, a group of compounds were screened for their efficacy in treating LX-2 cells at a concentration of 10 μM. This was evaluated by measuring the expression levels of α-SMA and collagen, the phosphorylation level of PKA, and the extent of inhibition of CRE promoter activity in cells (Data not shown). From this screening, the adenine derivative RAD11 (**Fig. 4A**) was selected as it demonstrated the highest potency among the compounds tested, including ZM241385 (data not shown). We also evaluated the potential non-specific toxicity of RAD11 using two normal cell lines, WI-38 (non-carcinogenic lung fibroblast cell line) and AML12 (non-carcinogenic liver hepatocyte cell line). RAD11 showed no substantial non-specific toxicity in normal cell lines (**Fig. S4**). We investigated the potential of RAD11 as a therapeutic agent for MASH-induced hepatic fibrosis by assessing its antifibrotic effects and its ability to function as a potent A2aAR antagonist. Using a CDAHFD-fed mouse model of MASH, we administered either RAD11 or ZM241385 at a dosage of 20 mg/kg every other day for 5 weeks (**Fig. 4B**). There were no significant differences in food intake (**Fig. 4C**) and body weight (**Fig. 4D**) between the RAD11- and ZM241385-administered groups throughout the experiment. The RAD11- and ZM241385-administered groups showed a similar reduction in the liver-to-body weight ratio (**Fig. 4E**). The RAD11-administered group demonstrated a more significant reduction in serum ALT and AST levels than the ZM241385-administered group (**Fig. 4F and G**). Hematoxylin and Eosin (H&E), Sirius red, and α-SMA staining results showed that RAD11 treatment reduced extensive accumulation of intrahepatic lipids and collagen in the liver increased by CDAHFD feeding, and α-SMA positive areas were more significantly reduced than with ZM241385 treatment. Myeloperoxidase (MPO) is known to accumulate more highly in livers with MASH 34, 35. Unlike in the ZM241385-treated group, MPO decreased significantly in the RAD11-administered group (**Fig. 4H and I**). Chemokine (C-X-C motif) ligand 1 (CXCL1) is reported to increase as MASH progresses due to increased MPO 35, 36. The level of CXCL1 was also increased in the livers of the CDAHFD group. RAD11 treatment reduced the CXCL1 levels more effectively than ZM241385 (**Fig. 4J**). The antifibrotic effect of RAD11 was further corroborated by a reduction in the protein levels of fibronectin and α-SMA (**Figs. 4K, L, and S5A**). In the CDAHFD group, mRNA levels of profibrotic markers, including *ACTA2*, *COL1A1*, *COL3A1*, *TIMP1*, and *PDGFB*, were significantly increased, but their expression was most significantly attenuated by RAD11 treatment (**Fig. 4M**). RAD11 demonstrated a more pronounced downregulation of A2aAR activity compared to ZM241385. This effect was observed through a greater decrease in c-AMP level and downregulation of A2aAR downstream factors in the RAD11-administered group, whereas the mRNA and protein levels of A2aAR remained consistent in all groups (**Figs. 4N-Q, and S5B**). Taken together, these findings indicate that RAD11 may hold great potential as a potent antifibrotic compound by alleviating hepatic fibrosis and inhibiting the A2aAR signaling pathway activated* in vivo* during the exacerbation of MASH and fibrotic progression induced by CDAHFD.

### RAD11 exerts antifibrotic effects in activated HSCs through effective inhibition of the A2aAR signaling pathway

**Figure 5:**
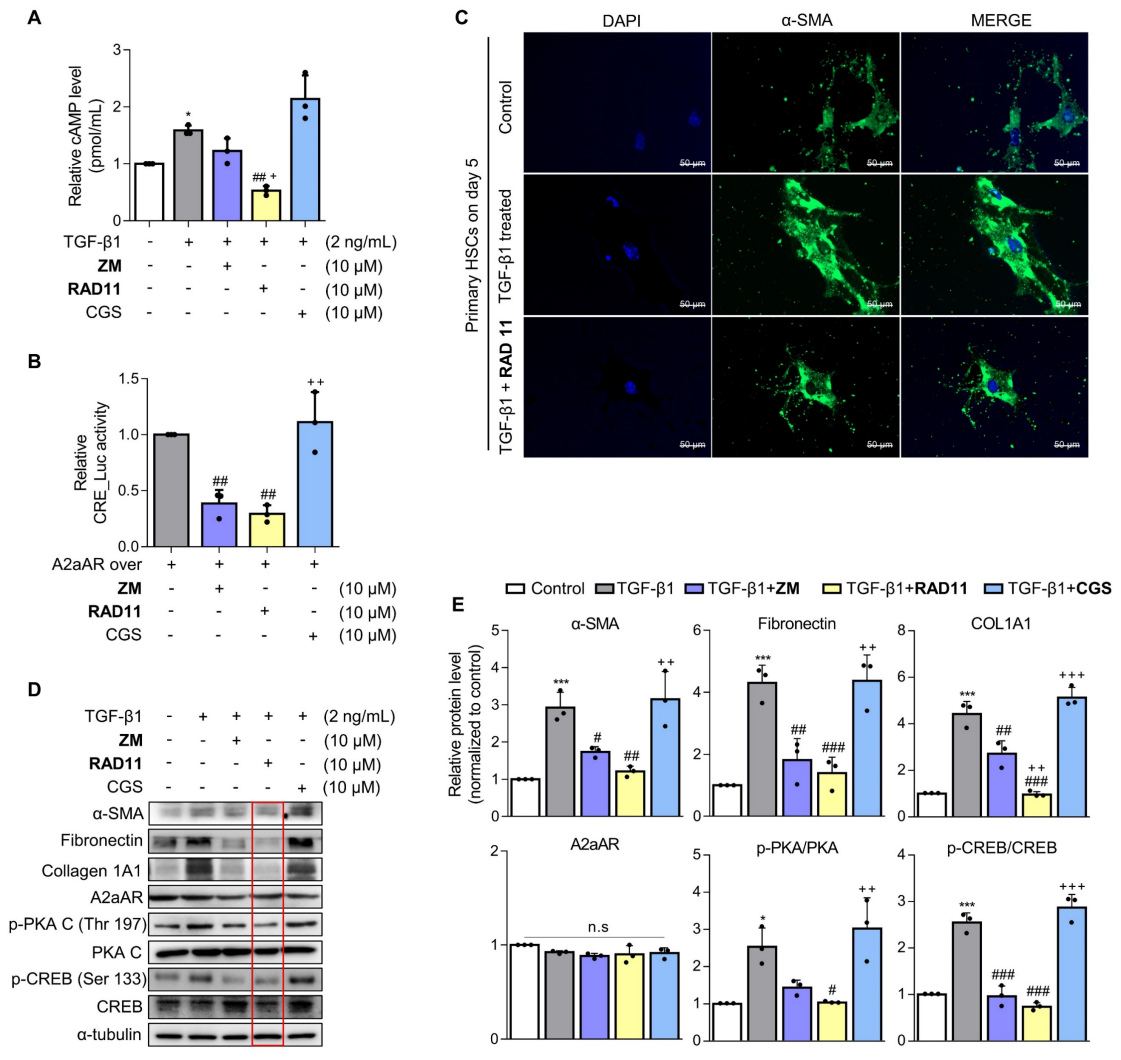
** RAD11 exerts antifibrotic effects in activated HSCs through effective inhibition of the A2aAR signaling pathway.** (A) Evaluation of total concentration of cAMP in LX-2 cells after treatment with 2 ng/mL TGF-β1 alone or in combination with ZM241385, CGS21680, or RAD11. (B) Measurement of CRE promoter activity after treatment with 10 μM ZM241385, CGS21680, or RAD11 for 18 h in LX-2 cells transfected with 0.5 μg pGL3-CRE alone or in combination with pcDNA4-myc-His A vector fused with hADORA2A vector. (C) Representative immunofluorescent images of primary HSCs treated with 2 ng/mL TGF-β1 alone or co-treated with 10 μM RAD11. Images were captured on day 5 after treatment with TGF-β1 alone or co-treatment with RAD11 (200× magnification). (D, E) Cultured LX-2 cells were activated by 2 ng/mL TGF-β1 for 6 h, followed by incubation with 10 μM of ZM241385, CGS21680, or RAD11 for 18 h. Collected cells were subjected to western blotting analyses. Statistical analyses were performed using ordinary one-way ANOVA. (A, B, E) *** *p* < 0.001, ** *p* < 0.01, * *p* < 0.05 versus control group; ### *p* < 0.001, ## *p* < 0.01, # *p* < 0.05 versus TGF-β1 alone-treated group; ⁺⁺⁺ *p* < 0.001, ⁺⁺ *p* < 0.01, ⁺ *p* < 0.5 versus ZM241385-treated group. n.s: nonsignificant.

To ascertain the mode of action of RAD11, LX-2 cells activated by TGF-β1 or A2aAR overexpression were treated with RAD11, ZM241385, or CGS21680. RAD11 reduced cAMP concentration and CRE promoter activity more effectively than ZM241385, but CGS21680 did not (**Figs. 5A and B**). Immunofluorescence images captured on day 5 of the primary HSC culture confirmed that RAD11 has antifibrotic activity. After treatment of TGF-β1, cultured HSCs exhibited elongated myofibroblast morphologies. This elongated pattern of activated HSCs was alleviated by treatment of RAD11 (**Fig. 5C**). These findings were consistent with observations in the mouse model of MASH (**Fig. 4**). Real-time video recording to monitor the activation of primary HSCs provided further support for the antifibrotic property of RAD11 (**Fig. S6**). The inhibition of activation events in primary HSCs by RAD11 was further corroborated in LX-2 cells.

RAD11 effectively blocked the fibrogenesis process and the A2aAR signaling pathway, as signified by reductions in protein levels of fibronectin, collagen 1A1, and downstream factors of activated A2aAR (**Figs. 5D and E**). To assess whether the RAD11-induced antifibrotic effect relies on A2aAR and its mediated signals, RAD11 was treated with the A2aAR-knockdowned or A2bAR-knockdowned LX-2 cells. RAD11 treatment did not induce the reduction in fibronectin and A2aAR-mediated p-PKA and p-CREB in the A2aAR-knockdowned cells (**Fig. S7A**). However, RAD11 treatment reduced the levels of fibronectin and A2aAR-mediated p-PKA and p-CREB, which were elevated by TGF-β1, in the A2bAR-knockdowned cells (**Fig. S7B**). These findings suggest that RAD11, similar to ZM241385, a known A2aAR-selective antagonist, induces PKA/CREB inhibition and antifibrotic effects through the A2aAR/PKA/CREB signaling axis, rather than the A2bAR/PKA/CREB, in HSC cells.

### The antifibrotic efficacy of RAD11 occurs through the reduction of lipid peroxidation and lipogenesis in hepatocytes

**Figure 6:**
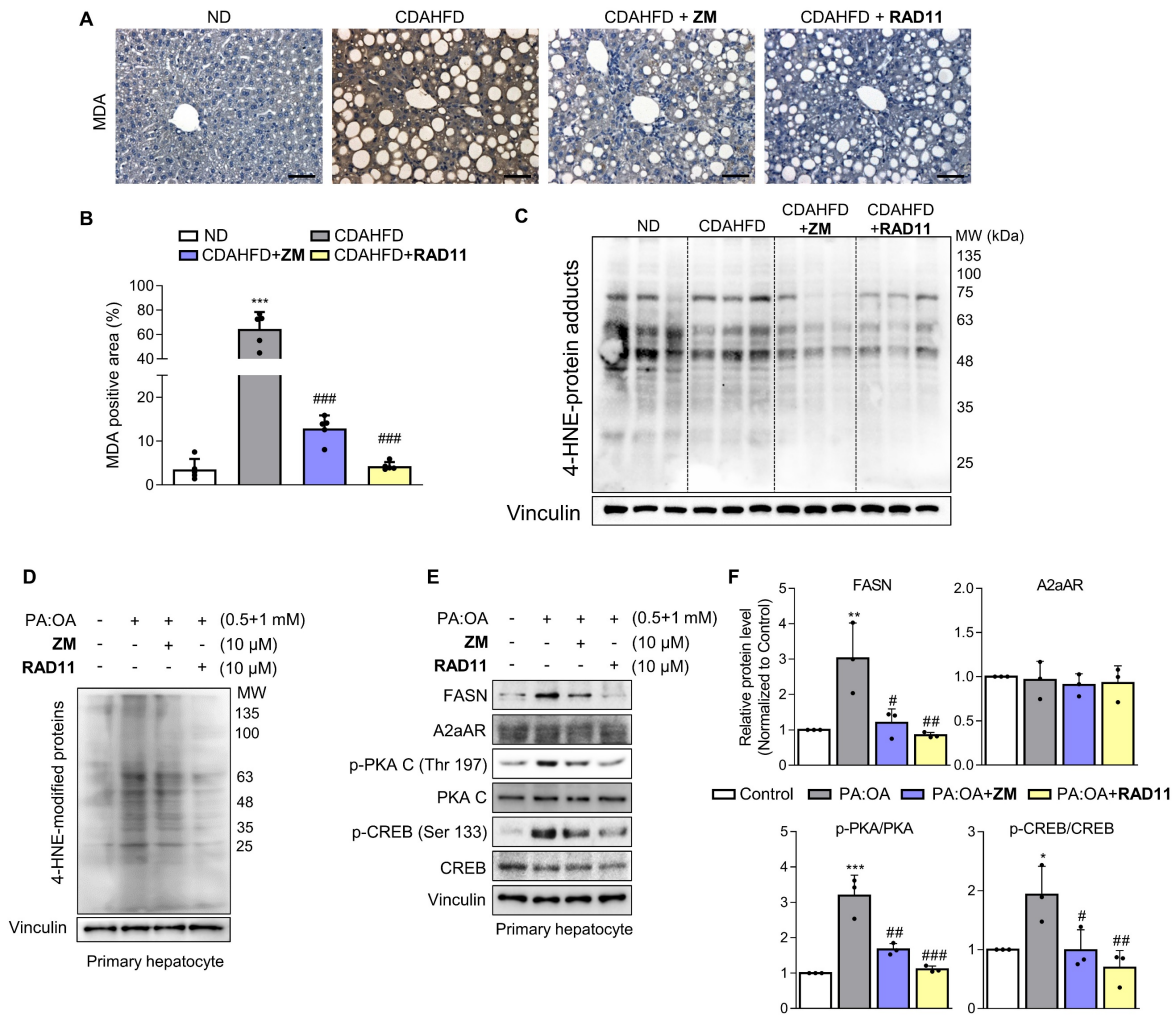
** The antifibrotic efficacy of RAD11 occurs through the reduction of both lipid peroxidation and lipogenesis in hepatocytes.** (A, B) Representative images and quantified graph of MDA staining of liver sections from each group (200× magnification. Scale bars, 10 μm). (C, D) The levels of 4-HNE-protein adducts were determined by western blot analysis using the liver tissues from each group and primary hepatocytes stimulated by PA:OA followed by treatment of each compound at concentration of 10 μM for 12 h. (E, F) Western blot analysis results and quantified graphs showing changes in the levels FASN, A2aAR itself, and A2aAR-mediated downstream signaling molecules in primary hepatocytes stimulated by PA:OA for 12 h, followed by treatment with 10 μM of ZM241385 or RAD11. PA:OA, a mixture of palmitic acid and oleic acid; FASN, fatty acid synthase. *** *p* < 0.001, ** *p* < 0.01, * *p* < 0.05 versus control group; ### *p* < 0.001, ## *p* < 0.01, # *p* < 0.05 versus PA:OA-treated group.

Upon observing augmented levels of 4-HNE-protein adducts caused by CDAHFD (**Fig. 1I**), we further assessed alterations in the levels of protein adducts resulting from the interaction of 4-HNE and malondialdehyde (MDA) after treatment with either ZM241385 or RAD11 (**Figs. 6A-C**). Compared to ZM241385, RAD11 reduced MDA- and 4-HNE-protein adducts more significantly. MDA- and 4-HNE-protein adducts are one of the final products of polyunsaturated fatty acids peroxidation in cells 37. To confirm this observation in hepatocytes, primary hepatocytes isolated from C57BL/6J mice were treated with PA:OA to emulate lipid accumulation-induced liver damage in MASH. Compared to ZM241385, RAD11 more efficiently reduced the increased 4-HNE-protein adducts induced by PA:OA (**Fig. 6D**). In addition, RAD11 attenuated the protein levels of fatty acid synthase (FASN), p-PKA, and p-CREB, which were increased by PA:OA treatment, more significantly than did ZM241385 (**Figs. 6E and F**). A2aAR protein expression was not changed by treatment with PA:OA alone or in co-treatment with RAD11 or ZM241385. Our results indicate that the antifibrotic action of RAD11 functions through a dual mechanism, effectively targeting both activated HSCs and hepatocytes. This leads to the suppression of HSCs and the reduction of lipogenesis in hepatocytes by inhibiting A2aAR-mediated signal cascades.

## Discussion

Adenosine functions as an endogenous modulator of tissue repair, particularly in situations involving cellular damage and tissue hypoxia. The generation of adenosine is facilitated by the dephosphorylation of adenosine triphosphates, diphosphates, and monophosphates 18, 38. Adenosine receptors belong to the superfamily of G-protein coupled receptors (GPCRs), and approximately 40% of the medications currently used target GPCRs to regulate a wide array of physiological processes 39. The signaling pathways governed by adenosine receptors present latent opportunities for mitigating the advancement of various diseases by modulating diverse signaling cascades through the distinct localization of each adenosine receptor 40. Notably, A2aAR has garnered considerable research attention due to its potential to ameliorate several conditions such as retinal 41, neurological 42, pulmonary 43, cardiac 44, and hepatic 45 disorders. Chan et al. offered compelling evidence substantiating the role of hepatic A2aAR in the progression of hepatic fibrosis in mice exposed to hepatotoxins such as TAA and CCl_4_. This discovery implies a plausible avenue for therapeutic intervention aimed at treating and thwarting liver cirrhosis 20-22. This revelation highlights the potential of A2aAR as a viable pharmacological target. However, there are substantial gaps in knowledge about the precise contribution of A2aAR to the complexity of hepatic fibrosis pathogenesis. MASH is a hepatic disorder characterized by abnormal buildup of triglycerides in liver cells. This condition leads to concurrent inflammation, cellular stress, and cell death 8-10, 46. MASH is histologically defined by the presence of hepatic steatosis, which involves the accumulation of lipid droplets within hepatocytes, accompanied by varying degrees of inflammation, hepatocyte ballooning, and fibrosis. Given the presence of these characteristics, MASH exhibits a wide range of pathological expressions, ranging from mild steatosis with negligible inflammation and absence of fibrosis to advanced cirrhosis 47. With the advancement of MASH, hepatic fibrosis is more frequent, representing a significant contributor to the increased liver-related mortality and complications 8-10, 48.

To explore the role of A2aAR signaling in the pathophysiology of hepatic fibrogenesis within the context of MASH, a mouse model of the disease was prepared in the present study using CDAHFD. The HFD-fed mice emulate the metabolic abnormalities of MASLD although it does not progress to advanced stages such as hepatic fibrosis and cirrhosis. In contrast, a methionine-choline-deficient diet (MCD) induces hepatic fibrosis in a mouse model within a span of 2 to 4 weeks, significantly impacting the expression of genes and proteins associated with lipid metabolism, inflammation, oxidative stress, and fibrogenesis pathways. This mirrors the advanced stage of human MASH. However, the MCD-fed model exhibits lower level of insulin resistance alongside substantial body weight loss, unlike the HFD-fed model. Consequently, the MCD-fed model does not effectively replicate the transition from MASLD (simple steatosis) to the advanced stage of MASH 49. Recent studies focusing on MASH coupled with hepatic fibrosis have employed a CDAHFD mouse model as a suitable experimental system 50-53. In the present study, we identified a higher level of *Adora2a* expression in HSCs compared to other cellular constituents in the liver. This observation is consistent with publicly available GSE34640 and GSE226103 datasets. No significant differences were observed in hepatic A2aAR protein level between CDAHFD-fed and standard ND-fed mice. This could be attributed to the analysis performed with the entire liver. However, the notable increase in the expression of p-PKA and p-CREB, recognized downstream effectors of A2aAR signaling, was observed in liver tissues collected from both MASH patients and mice with CDAHFD-induced MASH and fibrosis. In addition, there was a substantial elevation in the levels of 4-HNE-protein adducts, HSC activation markers, and profibrotic markers in liver tissues of CDAHFD-fed mice. This increase of profibrotic markers was confirmed in A2aAR-overexpressed LX-2 cells, similar to that observed in LX-2 cells treated with TGF-β1. These outcomes further reinforce the concept that the activation of HSCs through A2aAR signaling might be a substantial contributory factor in the pathogenesis of hepatic fibrosis induced by a CDAHFD. GSEA results also revealed that the cAMP/PKA/CREB signaling pathway-related gene set was more highly enriched in the MASH patients compared to the healthy group. Based on the results that ZM241385 treatment, but not CGS21680, reversed the TGF-β1-induced increase in profibrotic markers, we sought to discover therapeutic candidates for the treatment of hepatic fibrosis in MASH conditions through inhibition of the A2aAR signaling pathway. We conducted a screening with an in-house chemical library, and 21 novel adenine derivatives, each possessing A2aAR antagonistic properties, were then synthesized. Among the compounds synthesized, RAD11 was selected for further investigation due to its remarkable ability to significantly reduce the expression of proteins involved in A2aAR downstream signaling and profibrotic markers in LX-2 cells (data not shown). The administration of ZM241385, a selective A2aAR antagonist, reduced the liver-to-body weight ratio, serum ALT and AST levels, HSC activation maker α-SMA, and fibrotic markers like collagen deposition and fibronectin in both the livers of CDAHFD mice and LX-2 cells treated with TGF-β1. This observation is consistent with the impact of ZM241385 in mouse liver exposed to toxic substances such as TAA and CCl_4_ 20, and in the activated HSCs exposed to acetaldehyde 21. The administration of RAD11 demonstrated superior therapeutic effectiveness compared to ZM241385 in mitigating the histological manifestations of MASH and hepatic fibrosis. This pronounced effect could be due to the more distinctive ability of RAD11 to alleviate the augmented accumulation of MPO, as well as MDA- and 4-HNE-protein adducts in the liver, caused by CDAHFD-induced MASH. The escalation of reactive aldehydes, particularly MDA and 4-HNE, along with the protein adducts formed by these aldehydes, known for possessing proinflammatory and profibrogenic properties, has been linked to the severity of liver damage in humans with alcoholic liver disease and MASH 37, 54. This correlation was affirmed within the livers of the CDAHFD group. The elevation of CXCL1 due to MPO intensified with worsening MASH 34, 35, 55 and was verified in the livers of the CDAHFD group. The more prominent reduction achieved by RAD11 in terms of the protein adducts formed by MDA and HNE, as well as MPO and CXCL1, suggests that RAD11 provides enhanced protection to the liver against oxidative stress instigated by lipid accumulation.

Throughout the progression of hepatic fibrosis, it is widely acknowledged that HSCs play a pivotal role. These cells undergo transformation into activated myofibroblasts, actively participating in the synthesis of extracellular matrix proteins, which significantly contribute to the development of fibrosis 56, 57. Upon activation, HSCs release various cytokines and chemokines, including TGF-β, platelet-derived growth factor, and connective tissue growth factor 9, 57. Given that HSCs are instrumental in driving MASH-driven fibrosis, the prospect of developing pharmacological agents that effectively impede their activation holds great potential for advanced MASH treatment 50. Hence, a comprehensive understanding of the underlying mechanisms governing HSC activation is essential for unraveling the pathogenesis of hepatic fibrosis and for devising efficacious antifibrotic therapies.

The liver comprises approximately 70-85% hepatocytes and approximately 30% non-parenchymal cells, with their dynamic interplay contributing to homeostasis regulation 58. In addition to the observed superior antifibrotic effects of RAD11 on HSCs compared to ZM241385, RAD11 exhibited better antioxidant activity in hepatocytes compared to ZM241385. These findings offer compelling evidence that administration of RAD11 holds beneficial roles in both HSCs and hepatocytes. The superior therapeutic effectiveness of RAD11 on hepatic fibrosis in CDAFHD-fed mice and fibrogenesis of activated HSCs can be attributed to its multifaceted actions. RAD11 demonstrates the capacity to hinder HSC activation by suppression of A2aAR-mediated signaling and inhibition of hepatic lipogenesis in hepatocytes, mitigating the liver damage caused by lipid accumulation-induced oxidative stress. However, despite these promising findings, further exploration is necessary to clarify interactions among hepatic cell types and/or the potential intercellular communication mediators associated with the actions of RAD11.

In summary, our study sheds light on the significance of A2aAR-associated signal pathways in hepatic fibrogenesis in the context of MASH in a CDAFHD-fed mouse model. Furthermore, we successfully demonstrated the antifibrotic potential of RAD11, a newly synthesized small-molecule A2aAR antagonist, in CDAFHD-fed mice. Notably, RAD11, as a novel therapeutic agent for MASH and hepatic fibrosis, exhibited superior efficacy in alleviating MASH-related pathological features and in exerting antifibrotic effects without non-specific toxicity. Despite considerable progress in understanding of the molecular and cellular mechanisms involved, no drugs have yet gained approval from the FDA or European Medicine Agency (EMA) for the specific treatment of MASH and hepatic fibrosis 59. In this context, our findings hold significance as they may potentially pave the way for the development of an innovative therapeutic agent aimed at ameliorating hepatic fibrosis induced by MASH.

## Supplementary Material

Supplementary methods, figures and tables.

## Abbreviations

ACTA2: actin alpha 2
CREB: cAMP response element-binding protein
CDAHFD: chlorine-deficient, L-amino acid-defined, high-fat diet
COL1A1: collagen type 1 alpha 1 chain
COL3A1: collagen type III alpha 1 chain
CXCL1: C-X-C motif chemokine ligand 1
FASN: fatty acid synthase
HSCs: hepatic stellate cells
MDA: malondialdehyde
MPO: myeloperoxidase
ND: normal chow diet
PDGFB: platelet-derived growth factor subunit B
PKA: protein kinase A
RAD11: (4-(6-(methylamino)-2-(phenylethynyl)-9H-purin-9-yl)phenol
α-SMA: α-smooth muscle actin
TGF-β1: transforming growth factor beta 1
TIMP1: TIMP metallopeptidase inhibitor 1
4-HNE: 4-Hydroxy-2-nonenal
