# Supplementary material for: A novel A2a adenosine receptor inhibitor effectively mitigates hepatic fibrosis in a metabolic dysfunction-associated steatohepatitis mouse model
Source: Int J Biol Sci. 2024 Mar 3;20(5):1855–70. doi: 10.7150/ijbs.92371 (PMC10929195; doi:10.7150/ijbs.92371)
Supplement: Supplementary file 1 — Supplementary methods, figures and tables. [file ijbsv20p1855s1.pdf]

Supplemental information

A novel A2a adenosine receptor inhibitor effectively mitigates hepatic fibrosis in a metabolic dysfunction-associated steatohepatitis mouse model

Seojeong Park,[1#] Seohui Hwang,[1#] Jingyang Sun, Kyung-Hwa Jeon,[1] Naeun Sheen,[1] Sumin Shin,[1] Tae Hyun Kim,[2] Young-Sun Lee,[3] Wonhyo Seo,[1*] Jae-Sang Ryu,[1*] and Youngjoo Kwon[1*]

Contents

Synthesis of RAD11

Scheme S1. Synthesis of **RAD11 (5)**

Materials and methods

In vivo immunohistochemistry assay

Western blot analysis

cAMP assay

Figure S1. ^{1}H-NMR Spectrum of **RAD11**

Figure S2. ^{13}C-NMR Spectrum of **RAD11**

Figure S3. A2aAR/PKA/CREB signaling pathway is activated in MASH patients.

Figure S4. Cytotoxicity of **RAD11**

Figure S5. RAD11 represented its antifibrotic and A2aAR antagonistic effect by reducing relevant markers in a MASH mouse model.

Figure S6. Effect of **RAD11** on primary HSCs activated by TGF-β1

Figure S7. RAD11 inhibits the PKA/CREB signaling pathway through A2aAR in HSC.

Table S1. Antibodies and others

Table S2. Sequence based reagents

Synthesis of RAD11

General Information. All reactions were performed in oven-dried glassware fitted with glass stoppers under positive pressure of Ar with magnetic stirring, unless otherwise noted. Air- and moisture-sensitive liquids and solutions were transferred via a syringe or stainless-steel cannula. TLC was performed on 0.25 mm E. Merck silica gel 60 F254 plates and visualized under UV light (254 nm) or by staining with cerium ammonium molybdenate (CAM), potassium permanganate ($KMnO_4$), ninhydrin, or p-anisaldehyde. Flash chromatography was performed on E. Merck 230–400 mesh silica gel 60. Medium-pressure liquid chromatography (MPLC) was performed on a prepacked column (silica gel, 10 μm) with a UV detector. Reagents were purchased from commercial suppliers, and used without further purification unless otherwise noted. Solvents were distilled from proper drying agents (CaH_2 or Na wire) under an Ar atmosphere at 760 mmHg. All moisture- and/or oxygen-sensitive solids were handled and stored in a glovebox under N_2. NMR spectra were recorded on an Agilent (Santa Clara, CA, USA) Unity 400 instrument or a Bruker (Fällanden, Switzerland) AVANCE NEO Nanobay 400 MHz NMR spectrometer system equipped at Ewha Drug Development Research Core Center at 24 °C. Chemical shifts are expressed in ppm relative to TMS (^{1}H, 0 ppm), $CDCl_3$ (^{1}H, 7.26 ppm; ^{13}C, 77.2 ppm), DMSO-*d*$_6$ (^{1}H, 2.50 ppm; ^{13}C, 39.5 ppm), $(CD_3)_2CO$ (^{1}H, 2.05 ppm; ^{13}C, 206.3, 29.8 ppm), and CD_3OD (^{1}H, 3.31 ppm; ^{13}C, 49.1 ppm); coupling constants are expressed in Hz. High-resolution mass spectra (HRMS) were obtained on an Agilent 6230 TOF LC/MS (Agilent Technologies, Santa Clara, CA, USA) equipped at Ewha Drug Development Research Core Center by electrospray ionization (ESI, TOF), electron ionization (EI, magnetic sector), chemical ionization (CI, magnetic sector), or fast atom bombardment (FAB, magnetic sector). Purity and retention time (R_t) were determined by analytical HPLC. The HPLC analysis was conducted using an Agilent HPLC system, which consisted of a G1311A Quat pump, a G1314A VWD detector, and a G1329A autosampler. Phenomenex Jupiter 4 μm Proteo 90 Å column 250 × 4.6 mm analytical reverse-phase column was used. Solvents were eluted at a flow rate of 1 mL/min. Elution was carried out with the following gradient: binary solvent system, H_2O (solvent A), CH_3CN (solvent B), 10% to 100% B for 20 min, 100% B for 13 min, 100% to 0% B for 2 min, stop time 35 min, and the detection of compounds was performed at a wavelength of 254 nm.

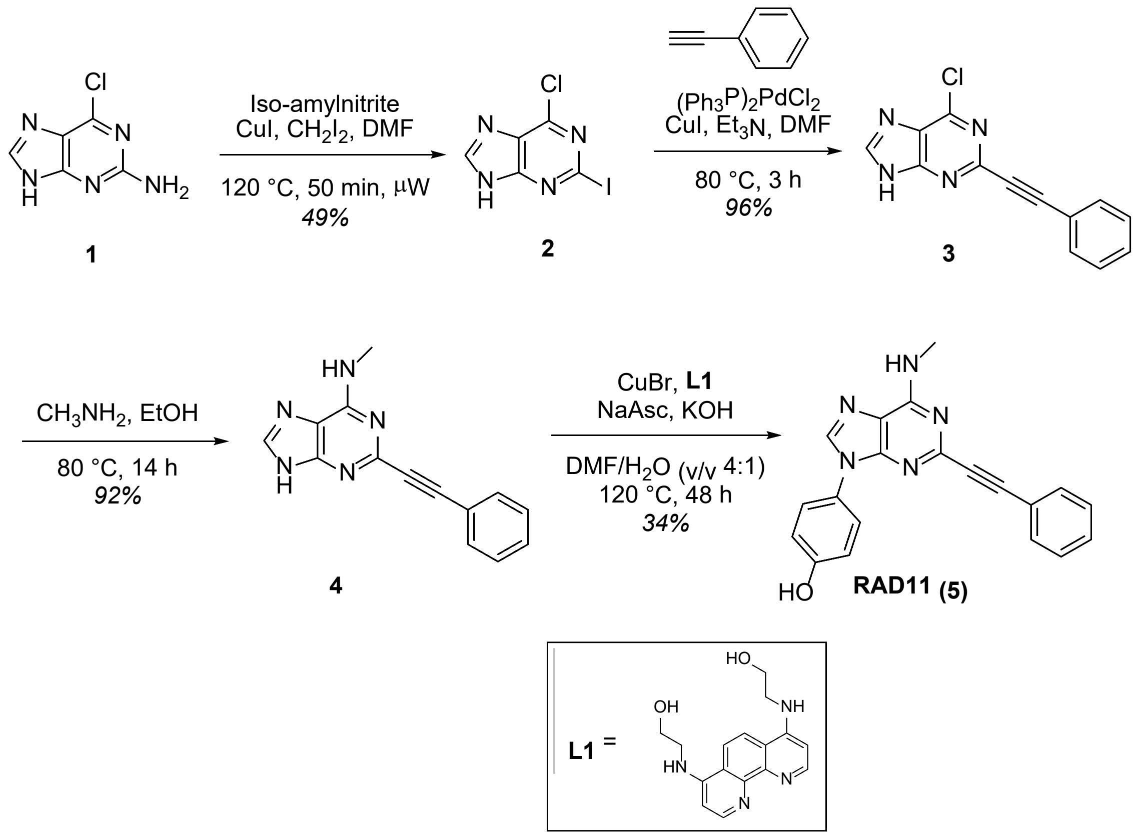

Scheme S1. Synthesis of **RAD11 (5)**.

6-Chloro-2-iodo-9*H*-purine (2)

In a 5 mL oven-dried round-bottom flask with a side arm, 2-amino-6-chloropurine (4.00 g, 23.6 mmol), CH_2I_2 (29.5 mL, 366 mmol), iso-amylnitrite (15.9 mL, 118 mmol) and CuI (449 mg, 2.36 mmol) were suspended in anhydrous DMF (12.0 mL). The resulting suspension was stirred at 120 °C for 50 min by microwave. Upon completion of the reaction, the reaction mixture was diluted with H_2O (200 mL) and extracted with EtOAc (3 × 200 mL). The combined organic extracts were dried over anhydrous $MgSO_4$, filtered, and concentrated in vacuo. The residue was purified by MPLC (1:2 hexane/EtOAc) to give iodopurine **2** as a yellow solid (3.27 g, 49%). TLC: R_f 0.45 (9:1 CH_2Cl_2/MeOH). Mp: 196.0−197.2 °C. ^{1}H NMR (400 MHz, DMSO-d_6): δ 14.00 (brs, 1H), 8.61 (s, 1H). ^{13}C NMR (100 MHz, DMSO-d_6) δ 155.2, 147.1, 146.6, 129.4, 117.4. HRMS (ESI) *m/z* calcd for $C_5H_3ClIN_4$ $[M + H]^+$ 280.9085, found 280.9086.

6-Chloro-2-(phenylethynyl)-9*H*-purine (3)

In a 50 mL oven-dried round-bottom flask with a side arm, 6-chloro-2-iodo-9*H*-purine (**2**) (3.27 g, 11.7 mmol), Et_3N (1.29 mL, 9.32 mmol), $(Ph_3P)_2PdCl_2$ (409 mg, 583 μmol), phenylacetylene (1.03 mL, 9.32

mmol) and CuI (111 mg, 583 μmol) were suspended in anhydrous DMF (20.0 mL). The mixture was stirred at 80 °C for 3 h. Upon completion of the reaction, the solvent was removed by rotary evaporation. The residue was purified by MPLC (1:1 hexane/EtOAc) to give alkynylpurine **3** as a yellow solid (2.84 g, 96%). TLC: R_f 0.30 (1:2 hexane/EtOAc). Mp: 165.8−166.9 °C. ^{1}H NMR (400 MHz, DMSO-d_6) δ14.05 (s, 1H), 8.76 (s, 1H), 7.69 (dd, J = 7.8, 1.7 Hz, 2H), and 7.56–7.46 (m, 3H). ^{13}C NMR (100 MHz, DMSO-d_6): δ 144.1, 132.1, 131.5, 131.4, 130.1, 129.0, 128.8, 128.7, 120.5, 88.0, and 85.9. HRMS (ESI) *m/z* calcd for $C_{13}H_8ClN_4$ ([M + H]$^+$) 255.0432, found 255.0433.

N-Methyl-2-(phenylethynyl)-9*H*-purin-6-amine (4)

In a 50 mL oven-dried round-bottom flask with a side arm, 6-chloro-2-(phenylethynyl)-9*H*-purine (**3**) (650 mg, 2.55 mmol) and methylamine (33 wt.% in EtOH, 1.91 mL, 15.3 mmol) were dissolved in anhydrous EtOH (7.5 mL). The resulting mixture was stirred at 80 °C for 24 h. Upon completion of the reaction, the solvent was removed by rotary evaporation. The residue was purified by MPLC (20:1 CH_2Cl_2/MeOH) to give aminopurine **4** as a yellow solid (587 mg, 92%). TLC: R_f 0.30 (20:1 CH_2Cl_2/MeOH). Mp: 232.5−233.9 °C. ^{1}H NMR (400 MHz, DMSO-d_6) δ 12.99 (s, 1H), 8.16 (s, 1H), 7.78 (s, 1H), 7.61 (dd, J = 6.7, 2.9 Hz, 2H), 7.51–7.39 (m, 3H), and 2.96 (s, 3H). ^{13}C NMR (100 MHz, DMSO-d_6): δ 154.7, 149.1, 145.3, 139.8, 131.8, 129.3, 128.9, 121.5, 118.6, 90.2, 82.4, and 27.1. HRMS (ESI) *m/z* calcd for $C_{14}H_{12}N_5$ ([M + H]$^+$) 250.1087, found 250.1087.

4-(6-(Methylamino)-2-(phenylethynyl)-9*H*-purin-9-yl)phenol (5) (RAD11)

In a reaction tube, *N*-methyl-2-(phenylethynyl)-9*H*-purin-6-amine (**4**) (100 mg, 401 μmol), CuBr (5.70 mg, 40.0 μmol), **L1** (23.9 mg, 80.0 μmol), NaAsc (15.8 mg, 80.0 μmol), KOH (45.0 mg, 802 μmol) and 4-bromophenol (104 mg, 602 μmol) were dissolved in DMF/H_2O (v/v = 4:1, 0.80 mL). The resulting mixture was stirred at 120 °C for 48 h. Upon completion of the reaction, the solvent was removed by rotary evaporation. The residue was purified by MPLC (50:1 CH_2Cl_2/MeOH) to give aminopurine **5** as a yellow solid (46 mg, 34%). Purity: 97% by HPLC. TLC: R_f 0.45 (30:1 CH_2Cl_2/MeOH). Mp: 253.3−254.6 °C. ^{1}H NMR (400 MHz, DMSO-d_6) δ 9.87 (s, 1H), 8.44 (s, 1H), 7.98 (s, 1H), 7.61 (dd, J = 7.3, 2.3 Hz, 2H), 7.58–7.50 (m, 2H), 7.52–7.40 (m, 3H), 7.00–6.89 (m, 2H), and 3.00 (s, 3H). ^{13}C NMR (100 MHz, DMSO-d_6): δ 157.2, 155.0, 148.4, 145.9, 140.9, 131.9, 129.5, 128.9, 126.1, 125.6, 121.3, 119.2, 115.9, 90.1, 83.0, and 27.2. HRMS (ESI) *m/z* calcd for $C_{20}H_{16}N_5O$ [M + H]$^+$ 342.1349, found 342.1352.

Materials and methods

In vivo immunohistochemistry assay

Liver tissues freshly excised from mice were immediately fixed in 4% paraformaldehyde and then used to prepare paraffin-embedded block sections for Hematoxylin and Eosin (H&E) staining, Sirius red staining, and immunohistochemistry (IHC). H&E staining was used to investigate liver histology and picrosirius red staining was used for hepatic fibrosis. Dewaxed and hydrated liver sections were stained with hematoxylin (# HMM999, ScyTek Inc. UT, USA), eosin (#318906, Sigma-Aldrich, MO, USA), and a mixture of picric acid solution (#ab150681, Abcam, Cambridge, UK), Fast-green FCF (Sigma Aldrich), and 0.1% direct red 80 (Sigma Aldrich) according to the manufacturers' instructions. The all-stained slides were evaluated at 100× magnification using the Axiophot 2 apparatus (Carl Zeiss MicroImaging Inc., Thornwood, NY, USA), equipped at Ewha Drug Development Research Core Center. Hepatic lipid accumulation area (%) and Sirius red-positive areas were quantified using Image J software (NIH, MD, USA). For IHC staining, dewaxed and hydrated liver sections were incubated with 3% hydrogen peroxide (Dako, Agilent, CA, USA) for 30 min and then blocked with serum buffer for 1 h. The primary antibody was applied and incubated overnight at 4°C, followed by incubation with the biotinylated secondary antibody for 30 min at room temperature. The slides were developed with ABC reagent solution (Vectastain Elite ABC kit, Vector Laboratories, CA, USA) for 30 min, covered with DAB solution (Dako, Agilent), and counterstained with Mayer's hematoxylin for 1 min. The IHC-stained slides were evaluated at 100× or 200× magnification using an Axiophot 2 apparatus (Carl Zeiss MicroImaging Inc.), and images were quantified using Image J.

Western blot analysis

Liver tissues were homogenized using TissueLyser II (QIAGEN, Hilden, Germany) in HEPES buffer containing 20 mM HEPES, 1% Triton X-100, 0.15 M NaCl, 1 mM EDTA, 10% glycerol, 1 mM EGTA, 5 mM NaF, a protease inhibitor cocktail, 0.5% NP-40, 1% PMSF, and 1 mM Na_3VO_4. LX-2 cells and primary hepatocytes were lysed in 1× RIPA lysis buffer (Cell Signaling, MA, USA) supplemented with 1 mM PMSF and 1% protease inhibitor cocktail. After centrifugation, the protein concentration of each sample was determined using the Pierce™ BCA assay kit (Thermo Fisher Scientific, MA, USA). The protein samples were then

subjected to SDS-PAGE, electro-transferred onto PVDF membranes, and non-specific sites on the membranes were blocked with 5% skim milk diluted with TBST. The membranes were then incubated with primary antibodies overnight at 4 °C, followed by incubation with horseradish peroxidase (HRP)-conjugated secondary antibodies. The interested blots were detected with an LAS-3000 (Fuji Photo Film Co., Ltd., Tokyo, Japan), equipped at Ewha Drug Development Research Core Center and analyzed using Multi Gauge 3.0 (Fuji Photo Film Co., Ltd., Tokyo, Japan) or Image Lab (Bio-Rad, CA, USA). Protein expression was quantified using Image J.

cAMP assay

LX-2 cells were seeded for 24 h, and the medium was replaced with a serum-free medium containing 2 ng/mL TGF-β1, followed by incubation for 6 h. After addition of 10 μM of either ZM241385, CGS21680, or compounds for 18 h, cells were lysed, and supernatants were collected for ELISA using a KGE002B kit (R&D Systems, MN, U.S.A). To measure cAMP concentration in the serum of each *in vivo* model, serum was diluted to one-fifth with Calibrator Diluent RD5-55. A primary antibody solution was added to the microplate, except for the non-specific binding well, and incubated at room temperature for 1 h, followed by addition of the cAMP conjugate. The standard, control, and sample were each added to individual wells and incubated for 2 h at room temperature. After incubation with substrate solution for 30 min, stop solution was added and optical density was measured at 450 nm. The absorbance at 540 nm was subtracted for optimal defect correction. Each sample was tested in triplicate, and the cAMP concentration was calculated using the mean absorbance obtained from the standard curve.

Gene set enrichment analysis (GSEA)

GSEA was conducted using the GSEA program (version 4.0.3) provided by the Broad Institute (http://www.broadinstitute.org/gsea/index.jsp). GSE63067 from the public Gene Expression Omnibus database (GEO; www.ncbi.nlm.nih.gov/geo) was analyzed to define the signature gene set.

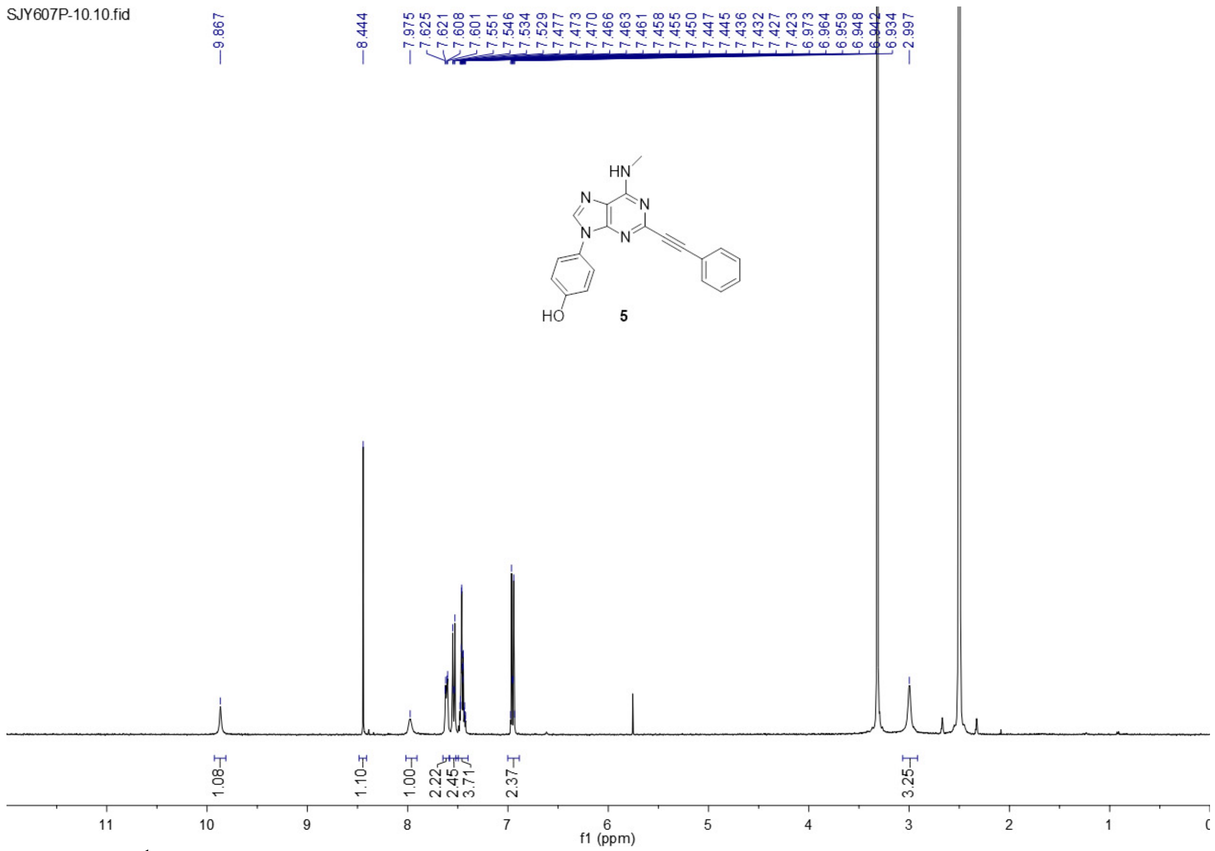

Figure S1. ^{1}H NMR (DMSO-d_6, 400 MHz) spectrum of **RAD11**

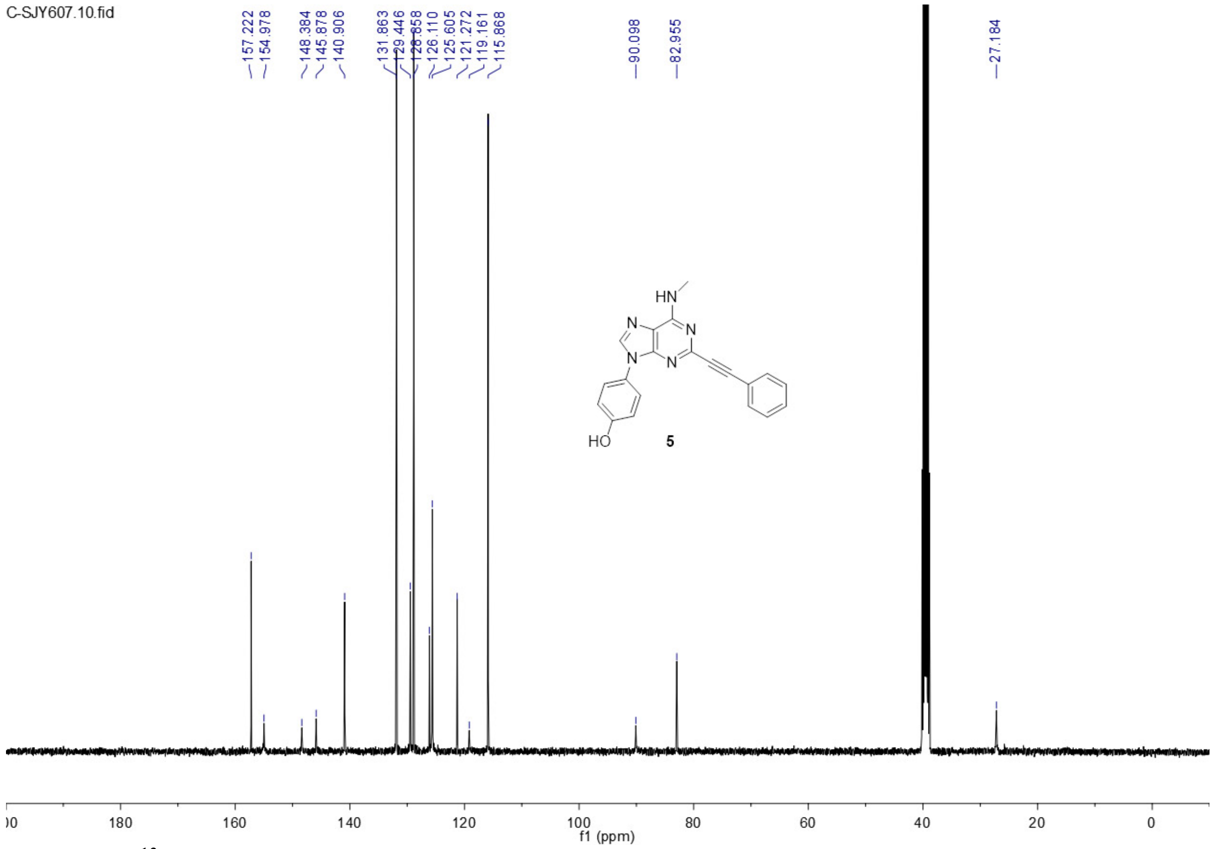

Figure S2. ^{13}C NMR (DMSO-d_6, 100 MHz) spectrum of **RAD11**

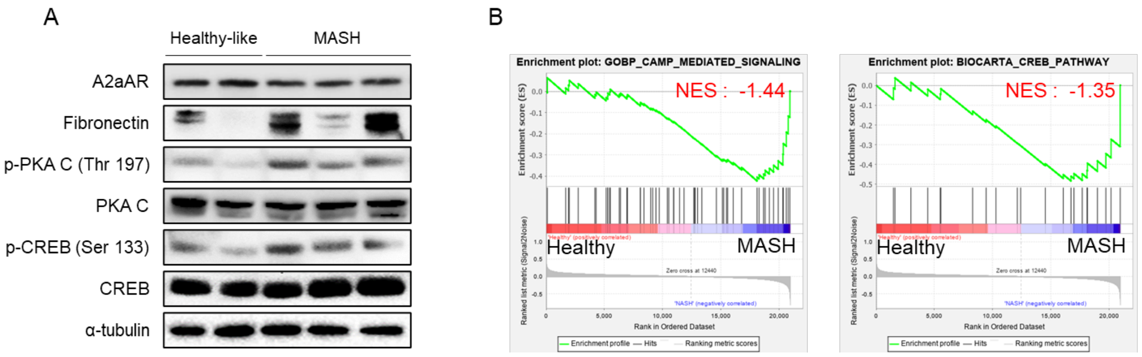

Figure S3. A2aAR/PKA/CREB signaling pathway is activated in MASH patients. To investigate the levels of p-PKA and p-CREB in patients with MASH, liver biopsy samples provided by Korea University Guro Hospital were categorized into two groups: a healthy-like group (n=2) with low METAVIR scores and a MASH group (n=3) with high METAVIR scores. This study was approved by the institutional review board of Korea University Guro Hospital (2016GR0302). All patients agreed collection of liver tissues and wrote informed consents by themselves. (A) Human liver biopsy samples were lysed and subjected to Western blot analyses. To investigate the levels of p-PKA and p-CREB, human biopsy samples were classified into healthy-like (n=2) and MASH groups (n=3) based on the METAVIR score. (B) GSE63067 for MASH patients was used for evaluating the gene set enrichment scoring, Healthy (n=7), MASH (n=9). NES: Normalized enrichment score, METAVIR: Meta-analysis of histological data in viral hepatitis.

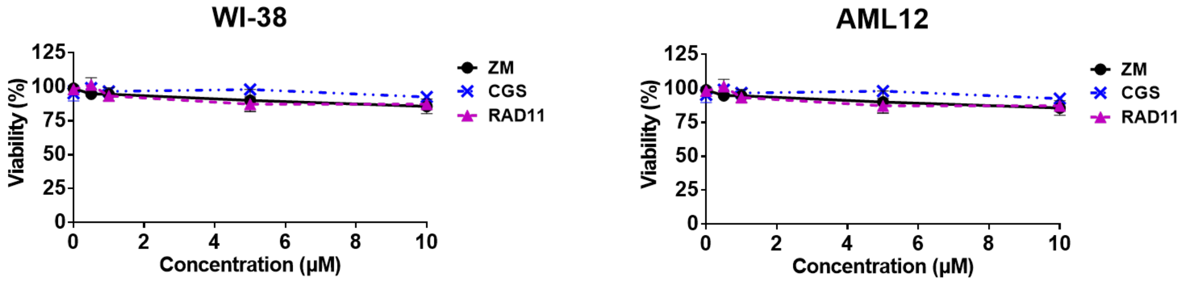

Figure S4. Assessment of RAD11 cytotoxicity on normal cells. In order to evaluate potential non-specific toxicity of RAD11, ZM241385, and CGS21680, each compound was treated for 24 h with two normal cell lines: WI-38 (non-carcinogenic lung fibroblast cell line) and AML12 (non-carcinogenic liver hepatocyte cell line). The compounds demonstrated no substantial non-specific toxicity within the normal cell lines.

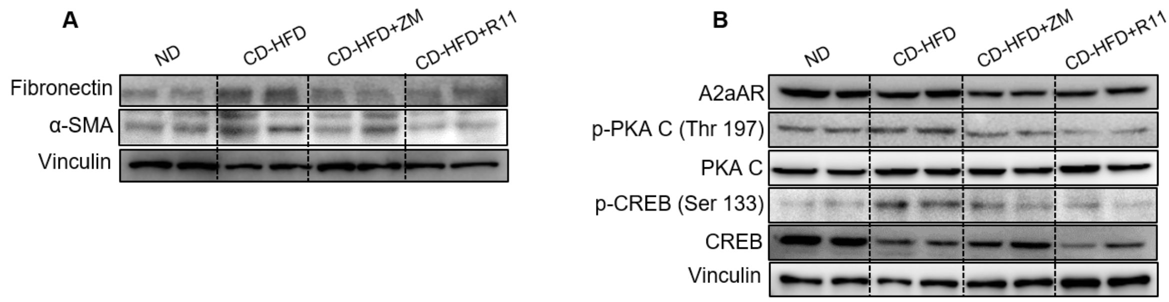

Figure S5. RAD11 represented its antifibrotic and A2aAR antagonistic effect by reducing relevant markers in a MASH mouse model. The protein expressions of fibrogenic markers (A) and A2aAR itself and its downstream factors (B) were analyzed by western blotting in each group. These were performed to meet the condition (n=5) in Fig. 4K and 4P, respectively.

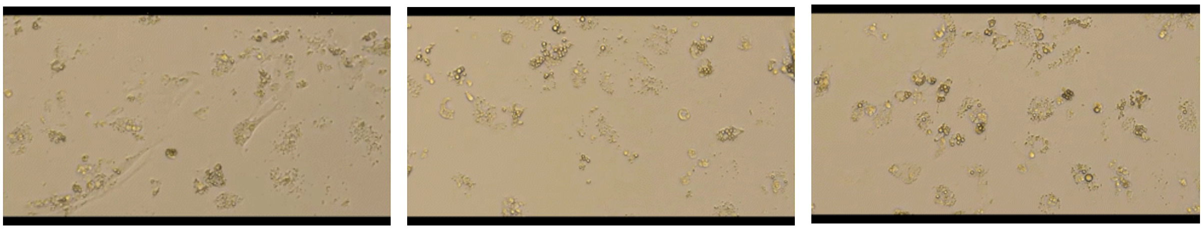

TGF- β1 TGF- β1+ZM241385 TGF- β1+R11

Figure S6. The effect of RAD11 on primary HSCs activated by TGF-β1. RAD11 was found to alleviate the degree of activation of primary HSCs induced by TGF-β1. To visualize the change in HSC morphology with administration of ZM241385 and RAD11, a video was recorded. Primary HSCs were isolated from C57BL/6J mice and treated with TGF-β1 only and co-treatment of TGF-β1 with either ZM241385 or RAD11 after 12 h of isolation.

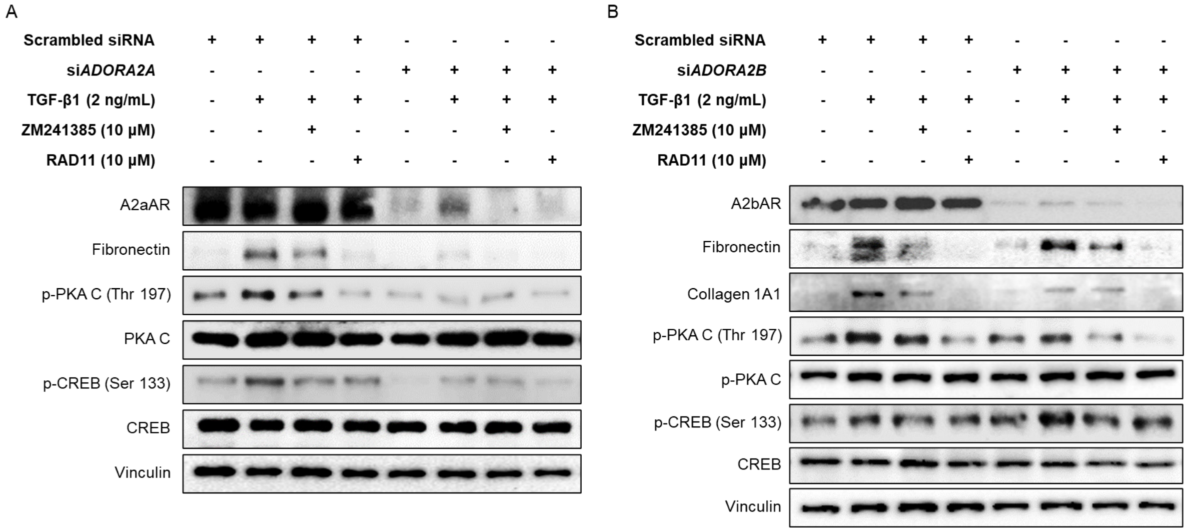

Figure S7. RAD11 inhibits the PKA/CREB signaling pathway through A2aAR in HSC. LX-2 cells were transfected with si*ADORA2A* (A) or si*ADORA2B* (B) for 24 h. Subsequently, the siRNA-treated LX-2 cells were pretreated with TGF-β1 for 6 h, followed by incubation with each compound for 18 h. All samples were then subjected to Western blot analysis.

Table S1. Antibodies and others

Name	Supplier	Cat no.
A2aAR	Abcam	ab3461
p-PKA C (Thr 197)	Cell signaling	#4781s
PKA C	Genetex	GTX104934
p-CREB (Ser 133)	Cell signaling	#9198s
CREB	Cell signaling	#9197s
α-SMA	Genetex	GTX100034
Fibronectin	Genetex	GTX112794
Collagen 1A1	Genetex	GTX112731
4-HNE	Cell Biolabs	STA-035
FASN	Cell signaling	#3189
Myeloperoxidase	Cayman Chemical	20493
Myc tag	Genetex	GTX628259
α-tubulin	MBL	M175-3
Vinculin	MBL	PM088
GAPDH	MBL	M171-3
Goat Anti-rabbit secondary ab (HRP)	Genetex	GTX213110-01
Anti-mouse IgG ab (HRP)	Genetex	GTX213111-01
Recombinant Human TGF-β1	R&D systems	7754-BH-005
ZM241385	Tocris	1036
CGS21680	Tocris	1063

Table S2. Sequence based reagents

Name	Sequence	Supplier
mACTA2	Fw: GTCCCAATCAGGGAGTAA<br>Rv: TCGGATACTTCAGCGTCAGGA	BIONICS
mCOL1A1	Fw: ATGGATCGTTCGAGTACG<br>Rv: TCAGCTGGATAGCGACATCG	BIONICS
mCOL3A1	Fw: CTGTAACATGGAAACTGGGGAAA<br>Rv: CCA TAGCTGAACTGAAAACCAC	BIONICS
mTIMP1	Fw: GCA ACT CGG ACC TGG TCA TAA<br>Rv: CGG CCC GTG ATG AGA AAC T	BIONICS
mPDGFB	Fw: GTG CTC GGG TCA TGT TCA AGT<br>Rv: CATCCGCTCCTTTGATGATCTT	BIONICS
mSOD1	Fw: CAGGACCTCATTTTAATCCTCAC<br>Rv: TGCCCAGGTCTCCAACAT	BIONICS
mSOD2	Fw: GACCCATTGCAAGGAACAA<br>Rv: GTAGTAAGCGTGCTCCCACAC	BIONICS
mCatalse	Fw: CCTTCAAGTTGGTTAATGCAGA<br>Rv: CAAGTTTTTGATGCCCTGGT	BIONICS
mCXCL1	Fw: TCTCCGTTACTTGGGGAC<br>Rv: CCACACTCAAGAATGGTCGC	BIONICS
mA2aAR	Fw: AACCTGCAGAACGTCACCA<br>Rv: GTCACCAAGCCATTGTACCG	BIONICS
mGAPDH	Fw: AATGGTGAAGGTCGGTGTG<br>Rv: GTGGAGTCATACTGGAACATGTAG	BIONICS
hCOL1A1	Fw: GGACACAGAGGTTTCAGTGG<br>Rv: CCAGTAGCACCATCATTTCC	BIONICS
hACTA2	Fw: AGTTACGAGTTGCCTGATGG<br>Rv: GAGGTCCTTCCTGATGTCAA	BIONICS
hGAPDH	Fw: CTTTGTCAAGCTCATTTCCTGG<br>Rv: TCTTCCTCTTGTGCTCTTGC	BIONICS
siADORA2A	Sense: CGACGACAGCUGAAGCAGA<br>Antisense: UCUGCUUCAGCUGUCGUCG	Bioneer Corp.
siADORA2B	Sense: CUGUGAAACAGUGUGAACU<br>Antisense: AGUUCACACUGUUUCACAG	Bioneer Corp.
